# Supplementary material for: Easy access to nucleophilic boron through diborane to magnesium boryl metathesis
Source: Nat Commun. 2017 Apr 7;8:15022. doi: 10.1038/ncomms15022 (PMC5385571; doi:10.1038/ncomms15022)
Supplement: Supplementary Information — Supplementary figures, supplementary tables, supplementary methods and supplementary references. [file ncomms15022-s1.pdf]

Supplementary Methods

General considerations and starting materials. All manipulations were carried out using standard Schlenk line and glovebox techniques under an inert atmosphere of argon. NMR experiments were conducted in Youngs tap NMR tubes made up and sealed in a Glovebox. NMR spectra were collected on a Bruker AV300 spectrometer operating at 300.2 MHz (^{1}H), 75.5 MHz (^{13}C), 96.3 MHz (^{11}B) or an Agilent ProPulse spectrometer operating at 500 MHz (^{1}H), 126 MHz (^{13}C), 160.4 MHz (^{11}B). The spectra were referenced relative to residual solvent resonances or an external $BF_3.OEt_2$ standard (^{11}B). Solvents (Toluene, THF, hexane) were dried by passage through a commercially available (Innovative Technologies) solvent purification system, under nitrogen and stored in ampoules over molecular sieves. C_6D_6 and d_8-toluene were purchased from Fluorochem Ltd. and Sigma-Aldrich Ltd.and dried over molten potassium before distilling under nitrogen and storing over molecular sieves. Di-*n*-butylmagnesium (1.0 M solution in *n*-heptane) and bis(pinacolato)diborane were purchased from Sigma-Aldrich Ltd. and used without further purification. [HC{(Me)CN(2,6-$^{i}Pr_2C_6H_3$)}$_2$Mg*n*Bu] (**7**) was synthesised by a literature procedure.[1] Elemental analyses were carried out at London Metropolitan University and at Elemental Microanalysis Ltd., Okehampton, Devon. UK.

Synthetic, spectroscopic and analytical data for new compounds

[HC{(Me)CN(2,6-$^{i}Pr_2C_6H_3$)}$_2$Mg{pinB(Bpin*n*Bu)}], (9)

In a J Youngs NMR tube, toluene (0.5 mL) was added to a mixture of compound **7** (50 mg, 0.1 mmol) and $(Bpin)_2$ (25.4 mg, 0.1 mmol). After 2 hours at room temperature, the NMR spectra showed the formation of a new complex (**9**). The solvent was removed under reduced pressure to afford compound **9** as a colourless solid (75.4 mg, 100%). Colourless crystals suitable for X-ray diffraction analysis were obtained from a saturated C_6D_6 solution at room temperature. Elemental analysis: Found C, 72.09; H, 9.61; N, 3.45%. $C_{45}H_{74}B_2MgN_2O_4$ requires: C, 71.78; H, 9.91; N, 3.72%. ^{1}H NMR (300 MHz, d_8-Tol): δ 7.11 (m, 6H, Ar-*H*), 4.80 (s, 1H, NC(CH$_3$)C*H*), 3.29 (hept, 2H, J_{HH} = 6.8Hz, C*H*(CH$_3$)$_2$), 3.19 (hept, 2H, J_{HH} = 6.8Hz, C*H*(CH$_3$)$_2$), 1.66 (s, 6H, NC(C*H*$_3$)CH), 1.34 (m, 24H, 8 x C*H*$_3$) 1.17 (d, 6H, JHH = 6.6 Hz, CH3), 1.11 (m, 12H, 4 x C*H*$_3$) 1.05 (d, 6H, JHH = 7.3 Hz, 2 x C*H*$_3$), 0.96 (m, 2H, C*H*$_2$) 0.89 (m, 2H, C*H*$_2$), 0.72 (s, 3H, CH$_2$-C*H*$_3$) -0.23 (m, 2H, C*H*$_2$-B) ppm. ^{13}C{^{1}H} NMR (75 MHz, d_8-Tol): δ 171.06 (N*C*(CH$_3$)CH), 146.23 (C_{ipso}), 143.16 (C_{ipso}), 142.84 (C_{ortho}), 142.41 (C_{ortho}), 126.41 (C_{para}), 126.25 (C_{para}), 125.06 (C_{meta}), 124.48 (C_{meta}), 95.01 (NC(CH$_3$)*C*H), 81.05 ((B(O*C*(CH$_3$)$_2$)$_2$), 78.80 ((B(O*C*(CH$_3$)$_2$)$_2$), 32.42,29.33,28.80, 28.40, 28.21 (*C*H(CH$_3$)$_2$), 27.42 (*C*H(CH$_3$)$_2$), 26.15 (CH$_2$-*C*H$_3$), 25.95 (C*H*$_2$-B), 25.82 (*C*H$_3$), 25.73 (*C*H$_3$), 25.69 (*C*H$_3$), 25.63 (*C*H$_3$), 25.43 (*C*H$_3$), 24.93 (NC(*C*H$_3$)CH), 23.48 (*C*H$_3$), 14.74 (*C*H$_3$) ppm. ^{11}B{^{1}H} NMR (96 MHz, d_8-Tol) : δ 37.25 (BuBpin), 10.40 (Bpin) ppm.

[HC{(Me)CN(2,6-^{i}Pr$_2$C$_6$H$_3$)}$_2$Mg{pinB}$_3$], (10)

A mixture of compound **7** (100.0 mg, 0.2004 mmol) and (Bpin)$_2$ (101.8 mg, 0.401 mmol) were dissolved in toluene (1 mL) and allowed to stir at room temperature. After 18 hours the solvent was removed under vacuum and the resultant white powder was washed with pentane (0.3 mL × 3) and dried under vacuum. Isolated yield 125.0 mg (0.152 mmol, 76%). Elemental analysis: Found: C, 68.63; H, 9.58; N, 3.55%. Calculated for $C_{47}H_{77}B_3MgN_2O_6$: C, 68.60; H, 9.43; N, 3.40%. ^{1}H NMR (C_6D_6, 298 K, 500.1 MHz): 7.25 (m, 2 H, Ar), 7.17 (m, 4 H, Ar), 4.82 (s, 1H, C*H*{C(CH$_3$)NDipp}$_2$), 3.34 (sep, 2H, ^{i}Pr-CH, $^3J_{HH}$ = 6.9 Hz), 3.25 (sep, 2H, ^{i}Pr-CH, $^3J_{HH}$ = 6.4 Hz), 1.68 (d, 3H, ^{i}Pr-CH$_3$, $^3J_{HH}$ = 6.4 Hz), 1.65 (s, 6H, CH{C(C*H*$_3$)NDipp}$_2$), 1.33 (d, 3H, ^{i}Pr-CH$_3$, $^3J_{HH}$ = 6.9 Hz), 1.29 (s, broad, 9H, Bpin-CH$_3$), 1.23 (d, 3H, ^{i}Pr-CH$_3$, $^3J_{HH}$ = 6.9 Hz), 1.18 (d, 3H, ^{i}Pr-CH$_3$, $^3J_{HH}$ = 6.4 Hz), 1.16 (s, 18H, Bpin-CH$_3$), 1.04 (s, 3H, Bpin-CH$_3$), 0.75 (s, broad, 6H, Bpin-CH$_3$). ^{13}C{^{1}H} NMR (C_6D_6, 298 K, 125.8 MHz): 171.1 (CH{*C*(CH$_3$)NDipp}$_2$), 145.3 (C^{1,2,6}-Ar), 144.1 (C^{1,2,6}-Ar), 142.5 (C^{1,2,6}-Ar), 125.2 (C^{3,4,5}-Ar), 124.5 (C^{3,4,5}-Ar), 123.9 (C^{3,4,5}-Ar), 95.3 (*C*H{C(CH$_3$)NDipp}$_2$), 83.4 (Bpin-*C*(CH$_3$)$_2$), 81.1 (Bpin-*C*(CH$_3$)$_2$), 79.3 (Bpin-*C*(CH$_3$)$_2$), 29.0 (^{i}Pr-CH), 28.9 (Bpin-C(*C*H$_3$)$_2$), 28.4 (^{i}Pr-CH), 26.2 (Bpin-C(*C*H$_3$)$_2$), 26.1 (^{i}Pr-CH$_3$), 26.1 (^{i}Pr-CH$_3$), 25.8 (Bpin-C(*C*H$_3$)$_2$), 25.6 (^{i}Pr-CH$_3$), 25.5 (Bpin-C(*C*H$_3$)$_2$), 25.5 (^{i}Pr-CH$_3$), 25.4 (CH{C(*C*H$_3$)NDipp}$_2$). ^{11}B NMR (C_6D_6, 298 K, 96.3 MHz): 37.5 (broad), 34.5 (broad), 5.3 (broad).

[HC{(Me)CN(2,6-^{i}Pr$_2$C$_6$H$_3$)}$_2$Mg(DMAP){Bpin}], (11)

In a J Youngs NMR tube, toluene (0.5 mL) was added to a mixture of compound **7** (200 mg, 0.4 mmol) and (Bpin)$_2$ (107 mg, 0.4mmol). After 2 hours, 1 equivalent of 4-dimethylaminopyridine (DMAP) (48.95 mg, 0.4 mmol) was added. The solvent was removed under reduced pressure and recrystallized from hexane at –35 °C to afford compound **11** as a colourless solid (188.3 mg, 68%). Colourless crystals suitable for X-ray diffraction studies analysis obtained from a saturated hexane solution of **11** at –35˚C. Elemental analysis: Found C, 72.17; H, 9.24; N, 8.07%. $C_{42}H_{63}B_1MgN_4O_2$ requires: C, 72.99; H, 9.19; N, 8.11%. ^{1}H NMR (300 MHz, d$_8$-Tol): δ 8.68 (br s, 2H,DMAP), 7.22 (m, 2H, Ar-*H*), 7.08 (m, 2H, Ar-*H*), 7.00 (m, 2H, Ar-*H*),6.00 (d, 2H, J_{HH} = 5 Hz, DMAP), 4.96 (s, 1H, NC(CH$_3$)C*H*), 3.74 (m, 2H, C*H*(CH$_3$)$_2$), 2.95 (m, 2H, C*H*(CH$_3$)$_2$), 2.19 (br s, 6H, N(C*H*$_3$)$_2$ DMAP), 1.80 (s, 6H, NC(C*H*$_3$)CH), 1.68 (d, 6H, J_{HH} = 6.2 Hz, CH(C*H*$_3$)$_2$), 1.35 (d, 6H, J_{HH} = 6.2 Hz, CH(C*H*$_3$)$_2$), 1.14 (d, 6H, J_{HH} = 6.2 Hz, CH(C*H*$_3$)$_2$), 0.96 (s, 12H, B(OC(C*H*$_3$)$_2$)$_2$), 0.55 (d, 6H, J_{HH} = 6.2 Hz, CH(C*H*$_3$)$_2$) ppm. (Free BuBpin 1.06 (s, 12H, B(OC(C*H*$_3$)$_2$)$_2$), 1.03 (s, 3H, C*H*$_3$-CH$_2$), 0.91 (m, 6H, (C*H*$_2$)$_3$CH$_3$) ppm). ^{13}C{^{1}H} NMR (75 MHz, d$_8$-Tol): δ 167.79 (N$\underline{C}$(CH$_3$)CH), 150.18 (aromatic $\underline{C}$H, ((CH$_3$)$_2$N$\underline{C}_5$H$_4$N)), 146.75($\underline{C}_{ipso}$), 143.39 ($\underline{C}_{ipso}$), 142.75 ($\underline{C}_{ipso}$), 125.06 ($\underline{C}_{para}$), 124.16 ($\underline{C}_{meta}$), 123.54 ($\underline{C}_{meta}$), 106.55 (aromatic $\underline{C}$H, ((CH$_3$)$_2$N$\underline{C}_5$H$_4$N)), 94.37 (NC(CH$_3$)$\underline{C}$H), 79.60 ((B(O$\underline{C}$(CH$_3$)$_2$)$_2$), 38.51 (($\underline{C}$H$_3$)$_2$NC$_5$H$_4$N), 29.59

($\underline{C}H(CH_3)_2$), 28.39 ($\underline{C}H(CH_3)_2$), 26.44 ($B(OC(\underline{C}H_3)_2)_2$), 25.69 ($\underline{C}H_3$), 25.41 ($\underline{C}H_3$), 25.03 ($\underline{C}H_3$), 24.57 ($\underline{C}H_3$), 24.37 ($\underline{C}H_3$), 23.49 ($NC(\underline{C}H_3)CH$) ppm. $^{11}B\{^1H\}$ NMR (160.44 MHz, d_8-Tol) :δ -5.40 (br s) ppm.

[HC{(Me)CN(2,6-$^iPr_2C_6H_3$)}$_2$Mg(DMAP)I], (12)

In a J Youngs NMR tube, 1 equivalent of iodomethane (6.23µL, 0.09 mmol) was added to a d_8-toluene (0.5 mL) solution of compound **11** (50 mg, 0.1 mmol), which induced the formation of a precipitate. The solvent was removed under reduced pressure to afford compound **12**, after washing with hexane, as a colourless solid in 71.6% yield. Colourless crystals suitable for X-ray diffraction studies were obtained by crystallisation from toluene. Elemental analysis: Found C, 63.08; H, 7.40; N, 7.69%. $C_{35}H_{51}N_4IMg$ requires: C, 62.57; H, 7.44; N, 8.11%. ^1H NMR (500 MHz, C_6D_6) δ 8.46 (d, 2H,DMAP), 7.22 (m, 1H, Ar-*H*), 7.12(s, 4H, Ar-*H*), 7.03 (m, 1H, Ar-*H*), 5.62 (d, 2H, DMAP), 4.99 (s, 1H, $NC(CH_3)C\mathit{H}$), 3.75 (sept, 2H, $C\mathit{H}(CH_3)_2$), 2.93 (sept, 2H, $C\mathit{H}(CH_3)_2$), 1.92 (s, 6H, $N(C\mathit{H}_3)_2$ DMAP), 1.77 (s, 6H, $NC(C\mathit{H}_3)CH$), 1.68 (d, 6H, $CH(C\mathit{H}_3)_2$), 1.29 (d, 6H, $CH(C\mathit{H}_3)_2$), 1.12 (d, 6H, $CH(C\mathit{H}_3)_2$), 0.61(d, 6H, $CH(C\mathit{H}_3)_2$) ppm. $^{13}C\{^1H\}$ NMR (126 MHz, C_6D_6) δ 169.01 ($N\underline{C}(CH_3)CH$), 163.85 (C IV), 149.00 (aromatic $\underline{C}H$, $((CH_3)_2N\underline{C}_5H_4N)$), 144.52 (aromatic $\underline{C}H$), 127.93 (aromatic $\underline{C}H$), 125.27 (aromatic $\underline{C}H$), 124.05 (aromatic $\underline{C}H$), 123.02 (aromatic $\underline{C}H$), 105.81 (aromatic $\underline{C}H$, $((CH_3)_2N\underline{C}_5H_4N)$), 93.99 ($NC(CH_3)\underline{C}H$), 37.75 ($(\underline{C}H_3)_2NC_5H_4N$), 28.94 ($\underline{C}H(CH_3)_2$), 27.86 ($\underline{C}H(CH_3)_2$), 24.78 ($\underline{C}H(CH_3)_2$), 24.71 ($\underline{C}H(CH_3)_2$), 24.45 ($\underline{C}H(CH_3)_2$), 24.03 ($NC(\underline{C}H_3)CH$), 23.54 ($\underline{C}H(CH_3)_2$). $^{11}B\{^1H\}$ NMR (96 MHz, d_8-Tol) 36.9 ppm.

[HC{(Me)CN(2,6-$^iPr_2C_6H_3$)}$_2$Mg{OCPh$_2$(Bpin)}], (13)

To a solution of compound **11** (20 mg, 0.029 mmol) in toluene(0.5 mL), in a J Youngs NMR tube, was added 0.9 equivalent of benzophenone (4.75 mg, 0.026 mmol). After 10 min the reaction was observed to be complete by ^1H NMR spectrsocopy. The solvent was removed under reduced pressure to give a pale green powder in 77 % yield (17.5 mg). Colourless crystals suitable for X-ray diffraction studies were obtained from a saturated solution of compound **13** in toluene. Elemental analysis: Found C, 74.91; H, 8.34; N, 5.93%. $C_{55}H_{73}BMgN_4O_3$ requires: C, 75.64; H, 8.43; N, 6.42%. ^1H NMR (500 MHz, d_8-Tol): δ 8.56 (d, 2H, J_{HH} = 5.6 Hz, DMAP), 7.30 (m, 4H, Ar-*H*), 7.21 (m, 4H, Ar-*H*), 7.11 (m, 2H, Ar-*H*), 6.99 (m, 6H, Ar-*H*), 5.93 (d, 2H, J_{HH} = 5.6 Hz, DMAP), 5.01 (s, 1H, $NC(CH_3)C\mathit{H}$), 3.65 (hept, 2H, J_{HH} = 6.5 Hz, $C\mathit{H}(CH_3)_2$), 2.96 (hept, 2H, J_{HH} = 6.5 Hz, $C\mathit{H}(CH_3)_2$), 2.14 (s, 6H, $N(C\mathit{H}_3)_2$ DMAP), 1.79 (s, 16H, $NC(C\mathit{H}_3)CH$), 1.31(m, 12H, $CH(C\mathit{H}_3)_2$) , 1.14 (d, 6H, J_{HH} = 6.5 Hz, $CH(C\mathit{H}_3)_2$), 0.93 (s, 12H, $B(OC(C\mathit{H}_3)_2)_2$), 0.62 (d, 6H, J_{HH} = 6.5 Hz, $CH(C\mathit{H}_3)_2$) ppm. $^{13}C\{^1H\}$ NMR (126 MHz, d_8-Tol): δ

168.34 ($N\underline{C}(CH_3)CH$), 152.84(C IV), 150.08 (aromatic $\underline{C}H$, $((CH_3)_2N\underline{C}_5H_4N)$), 146.55 (C IV), 142.47 (C IV), 141.84 (C IV), 127.79 (aromatic $\underline{C}H$), 126.98 (aromatic $\underline{C}H$), 124.57 (aromatic $\underline{C}H$), 123.99 (aromatic $\underline{C}H$), 123.74 (aromatic $\underline{C}H$), 123.14 (aromatic $\underline{C}H$), 105.27 (aromatic $\underline{C}H$, $((CH_3)_2N\underline{C}_5H_4N)$), 93.71 ($NC(CH_3)\underline{C}H$), 82.14 ($B(O\underline{C}(CH_3)_2)_2$), 37.70 ($(\underline{C}H_3)_2NC_5H_4N$), 28.66 ($\underline{C}H(CH_3)_2$), 27.75 ($\underline{C}H(CH_3)_2$), 24.93 ($CH(\underline{C}H_3)_2$), 24.49 ($CH(\underline{C}H_3)_2$), 24.35 ($CH(\underline{C}H_3)_2$), 24.23 ($B(OC(\underline{C}H_3)_2)_2$), 24.21 ($NC(\underline{C}H_3)CH$), 23.79 ($CH(\underline{C}H_3)_2$), 22.70 ($CH(\underline{C}H_3)_2$) ppm. $^{11}B\{^1H\}$ NMR (160 MHz, d_8-Tol): δ -4.14 ppm.

[HC{(Me)CN(2,6-^{i}Pr$_2$C$_6$H$_3$)}$_2$Mg{*i*-PrNC(Bpin)N*i*-Pr}(DMAP)], (14)

To a solution of compound **11** (20 mg, 0.029 mmol) in toluene (0.5 mL), in a J Youngs NMR tube, was added 0.9 equivalents of diisopropylcarbodiimide (4.43µL, 0.026 mmol). After 10 min the reaction is complete by ^1H NMR spectroscopy. The solvent was removed under reduced pressure to give a colourless powder in 85 % yield (18.4 mg). Colourless crystals of compound **14** suitable for X-ray diffraction analysis were obtained from a saturated solution of in hexane at -35˚C. Elemental analysis: Found C, 71.69; H, 9.62; N, 9.86%.$C_{49}H_{77}BMgN_6O_2$ requires: C, 72.01; H, 9.50; N, 10.28 %. ^1H NMR (500 MHz, toluene) δ 8.23 (br s, 2H,DMAP), 7.17- 6.99 (m, 6H, Ar-H), 5.99 (br s, 2H,DMAP), 4.91 (s, 1H, $NC(CH_3)C\mathit{H}$), 3.56 (br m, 6H, , $C\mathit{H}(CH_3)_2$), , 2.22 (s, 6H, $N(C\mathit{H}_3)_2$ DMAP), 1.72 (s, 6H, $NC(C\mathit{H}_3)CH$), 1.38 (d, 12H, $C\mathit{H}_3$), 1.32 (d, 12H, $C\mathit{H}_3$) 1.03 (m, 24H, $C\mathit{H}_3$). $^{13}C\{^1H\}$ NMR (126 MHz, toluene) δ 167.87 ($N\underline{C}(CH_3)CH$), 149.52 (aromatic $\underline{C}H$, $((CH_3)_2N\underline{C}_5H_4N)$), 146.91 ($C^{IV}$), 142.85 ($C^{IV}$), 128.76 (aromatic CH), 127.82 (aromatic CH), 124.98 (aromatic CH), 124.43 (C^{IV}), 123.46 (aromatic CH), 106.07 (aromatic $\underline{C}H$, $((CH_3)_2N\underline{C}_5H_4N)$), 95.30 ($NC(CH_3)\underline{C}H$), 83.44 ($(B(O\underline{C}(CH_3)_2)_2$), 48.47 ($N\underline{C}H(CH_3)_2$), 37.71 ($(\underline{C}H_3)_2NC_5H_4N$), 27.60 ($\underline{C}H(CH_3)_2$), 26.64 ($\underline{C}H_3$), 25.06 ($\underline{C}H_3$), 24.61 ($\underline{C}H_3$), 24.36 ($\underline{C}H(CH_3)_2$), 24.31 ($\underline{C}H_3$), 22.69 ($\underline{C}H_3$) ppm. $^{11}B\{^1H\}$ NMR (160 MHz, d_8-Tol): δ -8.17 ppm.

Supplementary Computational Methods

DFT calculations were run with Gaussian 09 (Revision D.01).[2] Mg and I centres were described with the Stuttgart RECPs and associated basis sets,[3] and 6-31G** basis sets were used for all other atoms (BS1).[4,5] Initial BP86[6,7] optimizations were performed using the 'grid = ultrafine' option, with all stationary points being fully characterized via analytical frequency calculations as either minima (all positive eigenvalues) or transition states (one negative eigenvalue). IRC calculations and subsequent geometry optimizations were used to confirm the minima linked by each transition state. All energies were recomputed with a larger basis set (BS2) featuring cc-pVTZ on I and 6-311++G** on all other atoms. Corrections for the effect of toluene (ε = 2.3741) solvent were run using the polarizable

continuum model and BS1.[8] Single-point dispersion corrections to the BP86 results employed Grimme's D3 parameter set with Becke-Johnson damping as implemented in Gaussian.[9]

Breakdown of Energy Contributions

The following tables detail the evolution of the relative energies as the successive corrections to the initial SCF energy are included. Terms used are:

**ΔE_{BS1}**	SCF energy computed with the BP86 functional with BS1
**ΔH_{BS1}**	Enthalpy at 0 K with BS1
**ΔG_{BS1}**	Free energy at 298.15 K and 1 atm with BS1
**$\Delta G_{BS1/tol}$**	Free energy corrected for toluene solvent with BS1
**$\Delta G_{BS1/tol+D3}$**	Free energy corrected for toluene and dispersion effects with BS1
**ΔG_{tol}**	Free energy corrected for basis set (BS2), dispersion effects and toluene solvent

In each case the final data used in the main article is highlighted in bold.

Energy Tables

Supplementary Table 1 Computed relative energies (kcal/mol) for the reactions of complexes **9** and **11**. Data in bold are those used in the main text. All energies are quoted relative to **9** at 0.0 kcal/mol.

	ΔE_{BS1}	ΔH_{BS1}	ΔG_{BS1}	$\Delta G_{BS1/tol}$	$\Delta G_{BS1/tol+D3}$	ΔE_{BS2}	ΔG_{tol}
**9**	0.0	0.0	0.0	0.0	0.0	0.0	**0.0**
**10**	-3.6	-3.5	-3.5	-1.9	-5.8	-0.6	**-2.8**
**11**	-14.4	-14.8	-22.1	-20.9	0.6	-15.1	**-0.1**
**12**	-86.2	-85.7	-96.1	-97.1	-75.6	-88.0	**-77.3**
**13**	-51.2	-50.4	-42.4	-40.5	-45.8	-49.6	**-44.2**
**14**	-47.0	-44.8	-26.6	-25.1	-54.3	-42.3	**-49.7**
**11**·IMe	-15.8	-15.5	-13.3	-11.5	0.9	-14.8	**1.9**
**TS(11-12)a**	-3.4	-3.5	1.5	0.5	5.4	-0.4	**8.4**
**INT(11-12)**	-67.2	-65.2	-59.2	-64.9	-65.3	-63.1	**-61.2**
**TS(11-12)b**	-59.7	-57.7	-49.2	-51.1	-56.3	-57.4	**-54.0**

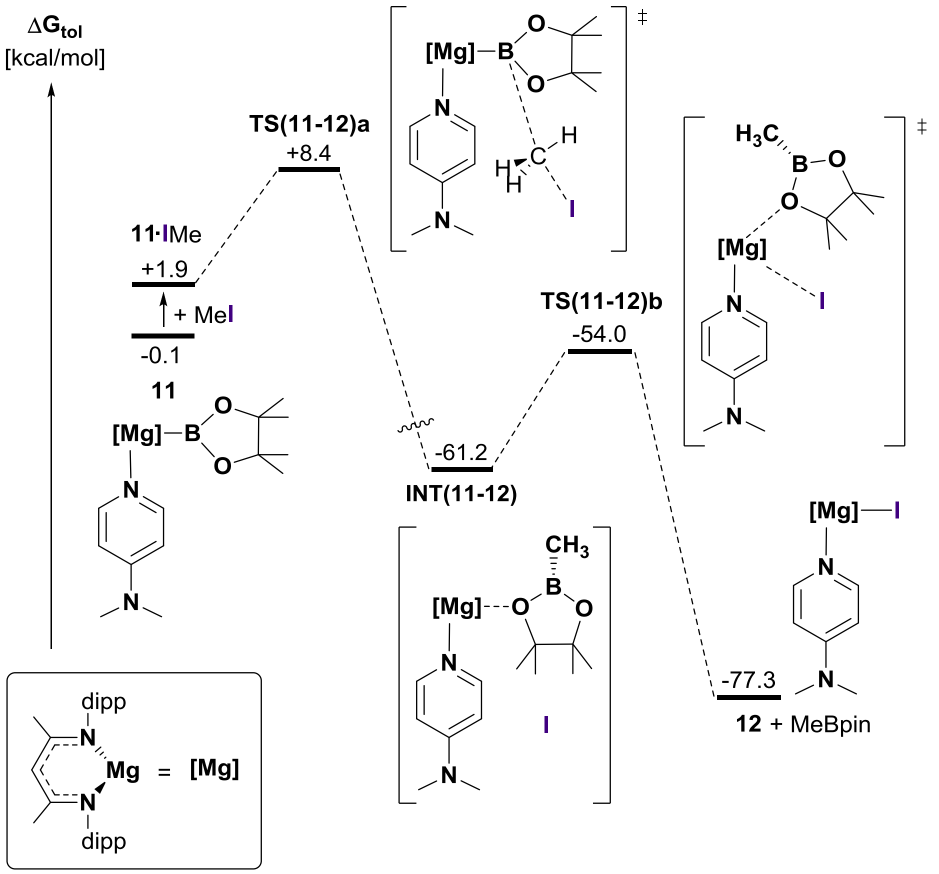

Supplementary Figure 1 DFT-calculated free energies (kcal/mol) relative to **9** for the addition of MeI to complex **11** in toluene.

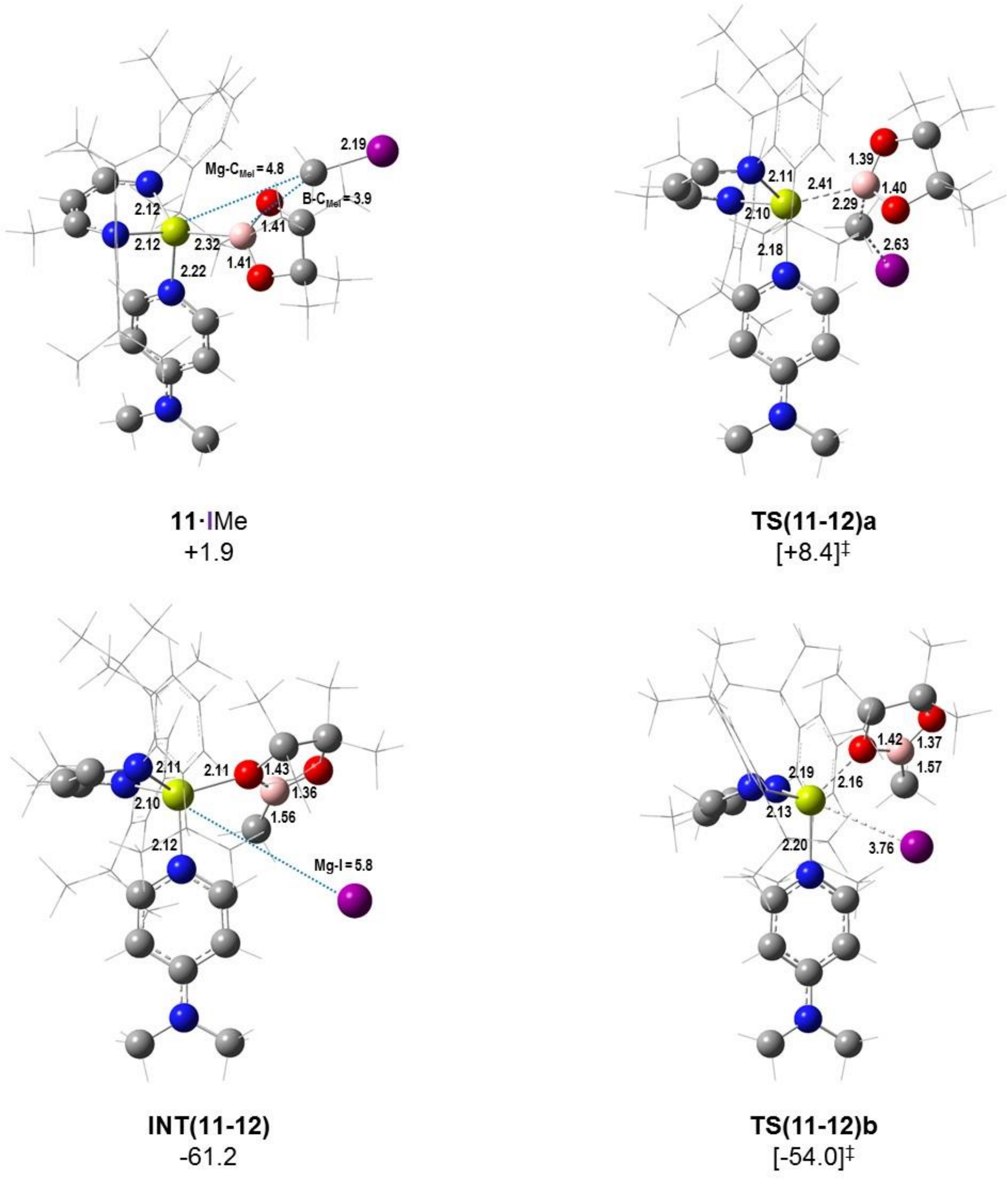

Supplementary Figure 2 DFT-computed geometries for the addition of MeI to complex **11**. Bond lengths given in Ångstroms.

Single Crystal X-ray Diffraction Analysis

Data were collected for compounds **9 - 14** on a SuperNova, Dual Cu at zero, EosS2 diffractometer. The crystal was kept at 150(2) K during data collection. Using Olex2,[10] the structures were solved via SHELXS[11] and refined with the SHELXL refinement package using Least Squares minimization. In compound **9** there is one molecule of benzene in the asymmetric unit, in addition to one molecule of the magnesium complex. H3 was located and refined at a distance of 0.95Å from C3. The asymmetric unit of compound **10** comprises one molecule of the magnesium complex, 1 full molecule of hexane and half of a hexane moiety which lies proximate to a crystallographic inversion centre that serves to generate the remainder. Some distance restraints were added to the refinement for the latter, to assist convergence. With the exception of C43, O5 and B1, all atoms in the latter ring fragment exhibited 50:50 disorder over 2 sites. This was modelled successfully, subject to some ADP restraints for C44 and C44A. ADP restraints were also included for C53. The asymmetric unit of compound **11** comprises 1 molecule of complex and 1 molecule of hexane. The BPin carbons were refined as exhibiting 60:40 disorder. There appeared to be some smearing of the electron density in this region and, hence, O-C, C-C and ADPs were refined subject to some restraints. In compound **12** there is ½ of a molecule of guest toluene present, proximate to an inversion centre, within the asymmetric unit of this structure. This necessarily means that the methyl group therein is disordered with a phenyl hydrogen therein. The asymmetric unit of compound **13** contains 2 molecules of the magnesium based complex, 2 half fragments of toluene (each proximate to a crystallographic inversion centre, which means that the methyl group is disordered over 2 sites in each case) plus a complete molecule of toluene in which the methyl group is disordered over 2 positions in a 75:25 ratio. Some distance restraints were added in the solvent regions as well as some ADP restraints in order to assist convergence. The phenyl ring in the full molecule of toluene was treated as a rigid hexagon. A further structure was determined during our initial study of the synthesis of compound **9**. This crystal, which was obtained from pentane solution, proved to be a co-crystal of compounds **9** and **10** (Figure S3). The asymmetric unit in this structure contains 2 different molecules and a region of solvent. In the species based on Mg1, the butyl group (C42-C45) was modelled as being disordered over 2 sites in a 65:35 ratio. Distance and ADP restraints were included for these fractional occupancy carbon atoms, to assist convergence. Likewise, in the moiety based on Mg2, all atoms in the pinacolborane based on B5 (with the exception of the boron atom itself) were disordered in a 50:50 ratio over 2 sites. As for the C42-C45 disorder, distance and ADP restraints were invoked in this region of electron density, to afford chemically sensible convergence. The solvent was largely evident as 2 molecules of pentane. However, the smearing of electron density associated with it would have required an over-paramaterized model to accommodate an associated disorder model. Thus, this electron density was ultimately treated with PLATON SQUEEZE, and an allowance for 2 molecules of C_5H_{12} was made in the formula of the asymmetric unit presented herein.

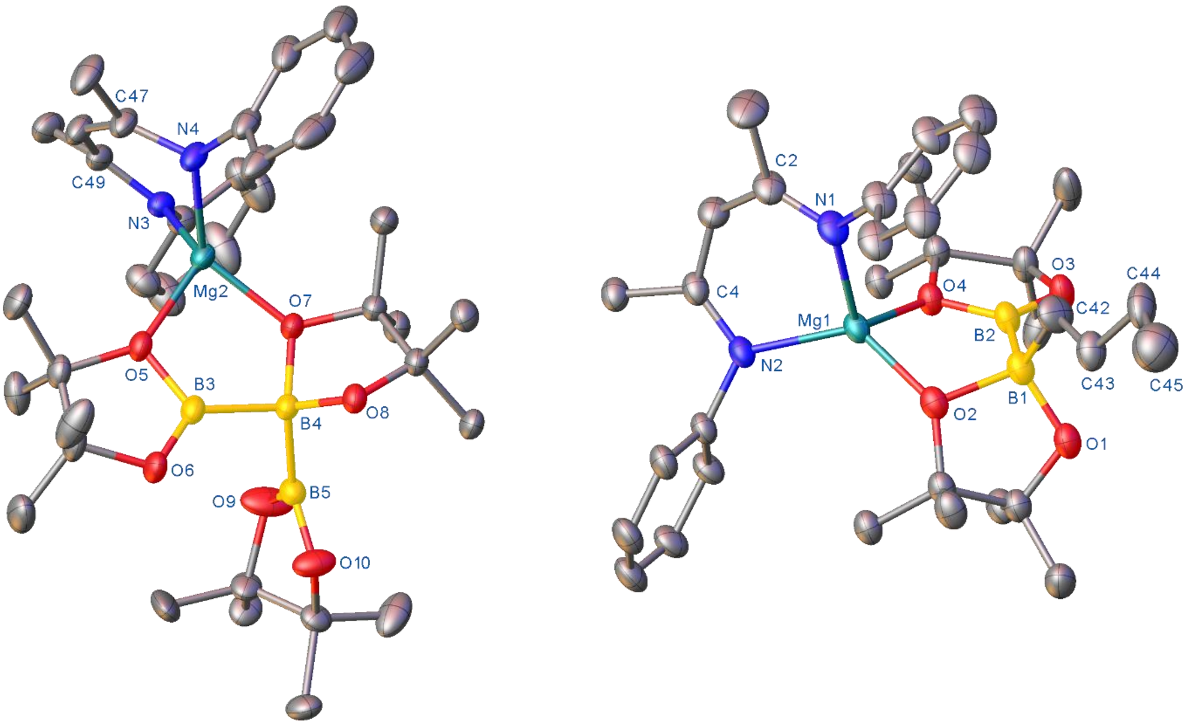

Supplementary Figure 3: The molecular structure obtained from a co-crystal of compounds **9** and **10**.

Supplementary Table 2 Single crystal X-ray diffraction analysis of compounds **9** – **14** and the co-crystal of **9** and **10**.

Compound	**9**	**10**	**11**	**12**	**13**	**14**	**9**/**10** (co-crystal)
Empirical formula	$C_{51}H_{80}B_2MgN_2O_4$	$C_{56}H_{98}B_3MgN_2O_6$	$C_{48}H_{77}BMgN_4O_2$	$C_{79}H_{110}I_2Mg_2N_8$	$C_{124}H_{162}B_2Mg_2N_8O_6$	$C_{49}H_{77}BMgN_6O_2$	$C_{102}H_{175}B_5Mg_2N_4O_{10}$
Formula weight	831.10	952.10	777.25	1474.16	1930.85	817.28	1720.12
Temperature/K	150.00(10)	150.00(10)	150.01(10)	150.01(10)	150.00(10)	150.00(10)	293(2)
Crystal system	monoclinic	monoclinic	orthorhombic	monoclinic	triclinic	monoclinic	triclinic
Space group	*P*2$_1$/*n*	*P*2$_1$/*c*	*Pbca*	*I*2/*a*	*P*-1	*P*2$_1$/*c*	*P*-1
a/Å	10.86110(10)	14.2104(3)	18.80242(12)	15.16946(13)	13.8498(4)	12.86959(13)	12.4093(5)
b/Å	26.61530(10)	34.8042(5)	16.94782(10)	15.25569(10)	20.5338(5)	16.72409(14)	21.7207(8)
c/Å	17.48810(10)	13.1173(2)	30.45101(18)	34.6966(3)	21.4903(5)	23.2522(3)	22.3714(6)
α/°	90	90.00	90	90	96.998(2)	90	67.492(3)
β/°	98.0240(10)	112.916(2)	90	98.0263(8)	101.818(2)	102.3406(10)	76.478(3)
γ/°	90	90.00	90	90	99.846(2)	90	88.860(3)
U/Å^3	5005.82(6)	5975.54(18)	9703.52(10)	7950.85(11)	5815.5(3)	4888.99(9)	5399.9(4)
Z	4	4	8	4	2	4	2
ρ_{calc}g/cm^3	1.103	1.058	1.063	1.232	1.103	1.110	1.058
μ/mm^{-1}	0.631	0.603	0.603	6.700	0.611	0.634	0.609
F(000)	1816.0	2092.0	3408.0	3080.0	2088.0	1784.0	1888.0
Crystal size/mm^3	0.324×0.235×0.176	0.1693×0.1183×0.0646	0.346×0.257×0.194	0.213×0.189×0.116	0.364×0.133×0.106	0.309×0.2×0.14	0.296×0.168×0.078
2θ range for data collection/°	6.09 to 146.934	8.2 to 139.52	7.472 to 147.002	6.34 to 146.81	5.622 to 144.97	6.564 to 146.25	7.35 to 148.014
Index ranges	$-11 \le h \le 13$, $-33 \le k \le 33$, $-21 \le l \le 21$	$-13 \le h \le 17$, $-38 \le k \le 42$, $-15 \le l \le 11$	$-23 \le h \le 23$, $-20 \le k \le 16$, $-37 \le l \le 37$	$-18 \le h \le 17$, $-18 \le k \le 16$, $-42 \le l \le 43$	$-17 \le h \le 16$, $-24 \le k \le 25$, $-18 \le l \le 26$	$-15 \le h \le 15$, $-20 \le k \le 14$, $-28 \le l \le 28$	$-15 \le h \le 11$, $-26 \le k \le 26$, $-27 \le l \le 27$
Reflections collected	77687	23248	138871	44200	47028	61543	82570
Independent reflections, R_{int}	10077 [R_{int} = 0.0371, R_{sigma} = 0.0194]	10974 [R_{int} = 0.0275, R_{sigma} = 0.0389]	9750 [R_{int} = 0.0406, R_{sigma} = 0.0140]	7971 [R_{int} = 0.0211, R_{sigma} = 0.0136]	22626 [R_{int} = 0.0222, R_{sigma} = 0.0352]	9778 [R_{int} = 0.0340, R_{sigma} = 0.0187]	21440 [R_{int} = 0.0858, R_{sigma} = 0.0605]
Data/restraints/parameters	10077/1/564	10974/18/690	9750/131/613	7971/0/428	22626/49/1321	9778/0/552	21440/68/1127
Goodness-of-fit on F^2	1.042	1.028	1.045	1.035	1.025	1.021	1.045
Final $R1$, wR_2[I>=2σ (I)]	R_1 = 0.0382, wR_2 = 0.0940	R_1 = 0.0697, wR_2 = 0.1872	R_1 = 0.0634, wR_2 = 0.1873	R_1 = 0.0295, wR_2 = 0.0815	R_1 = 0.0449, wR_2 = 0.1173	R_1 = 0.0379, wR_2 = 0.0999	R_1 = 0.0898, wR_2 = 0.2690
Final $R1$, wR_2[all data]	R_1 = 0.0415, wR_2 = 0.0964	R_1 = 0.0877, wR_2 = 0.2039	R_1 = 0.0668, wR_2 = 0.1914	R_1 = 0.0303, wR_2 = 0.0821	R_1 = 0.0605, wR_2 = 0.1268	R_1 = 0.0436, wR_2 = 0.1040	R_1 = 0.1234, wR_2 = 0.3137
Largest diff. peak/hole / e Å^{-3}	0.31/-0.24	0.82/-0.36	0.56/-0.63	1.01/-1.06	0.41/-0.41	0.26/-0.18	0.70/-0.72

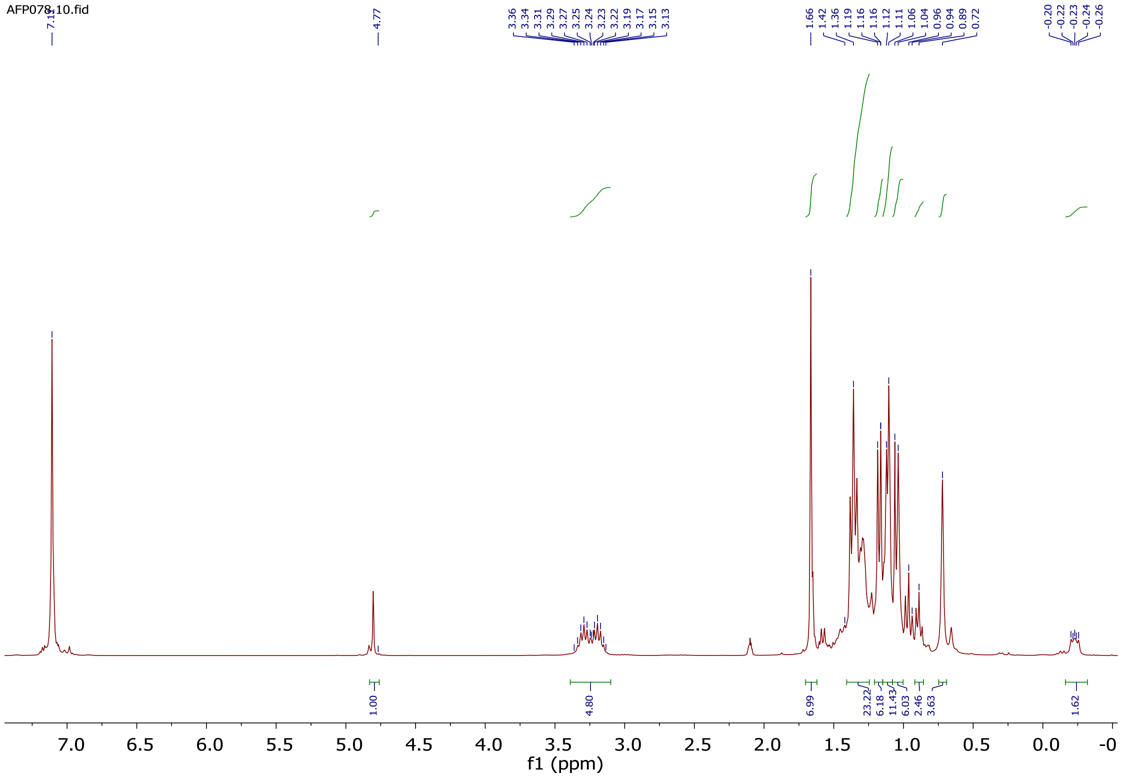

Supplementary Figure 4: ^{1}H NMR spectrum of **9** in d_8-Toluene

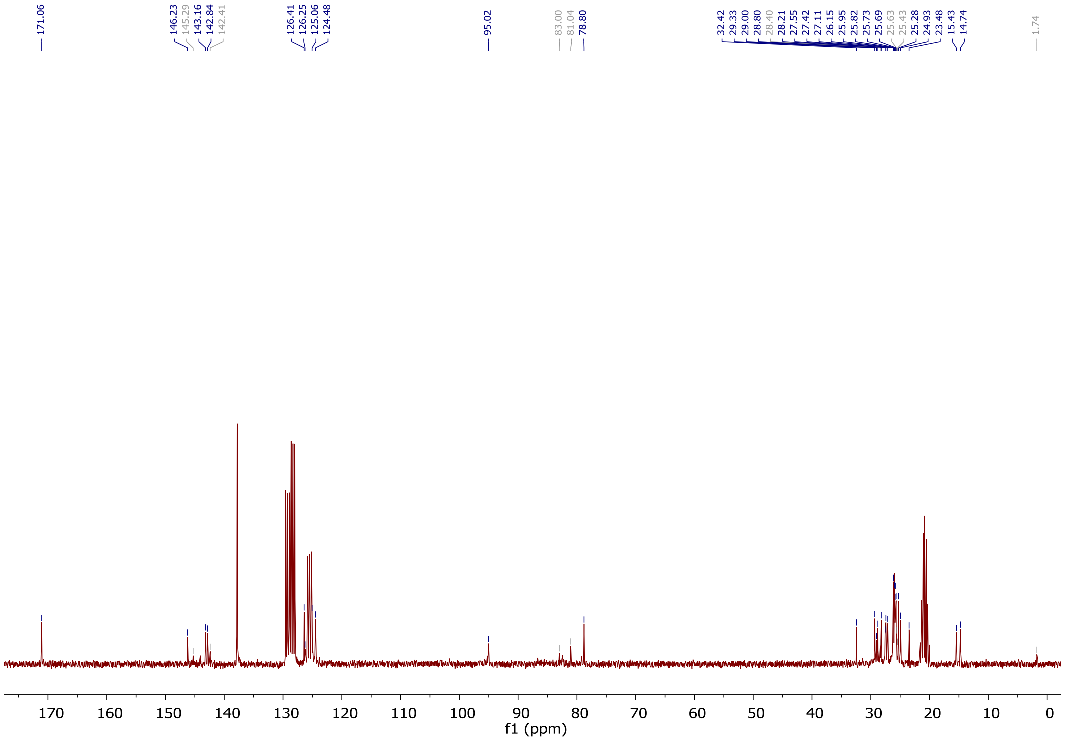

Supplementary Figure 5: ^{13}C{^{1}H} NMR spectrum of **9** in d_8-Toluene

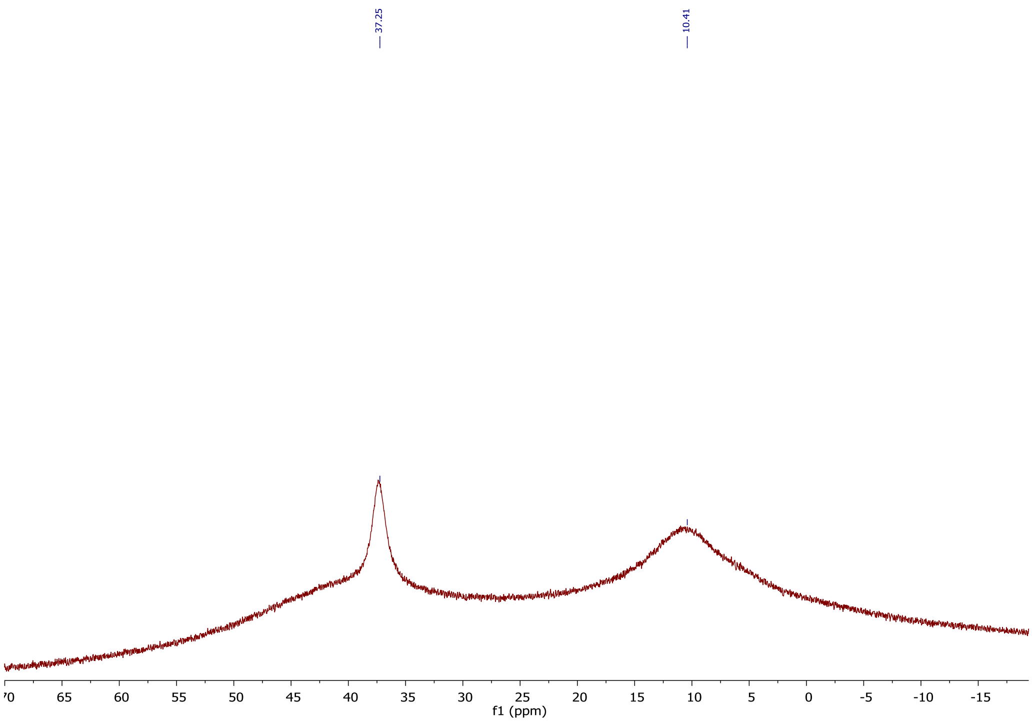

Supplementary Figure 6: $^{11}B\{^{1}H\}$ NMR spectrum of **9** in d_8-Toluene

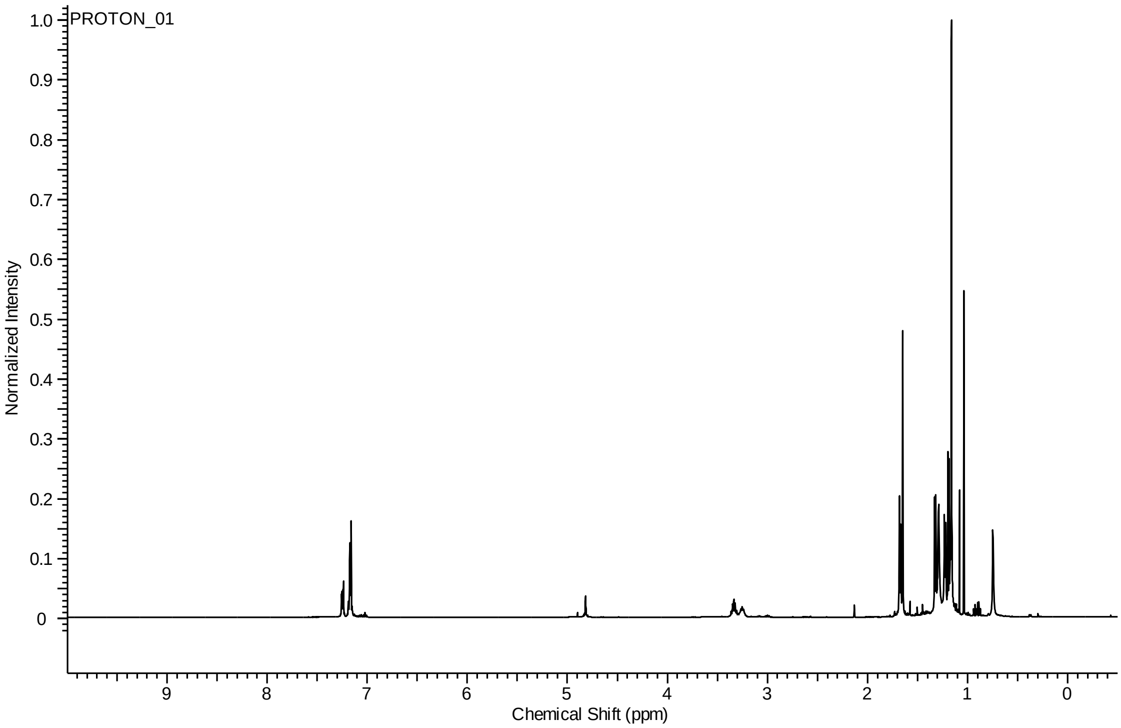

Supplementary Figure 7: ^{1}H NMR spectrum of **10** in C_6D_6

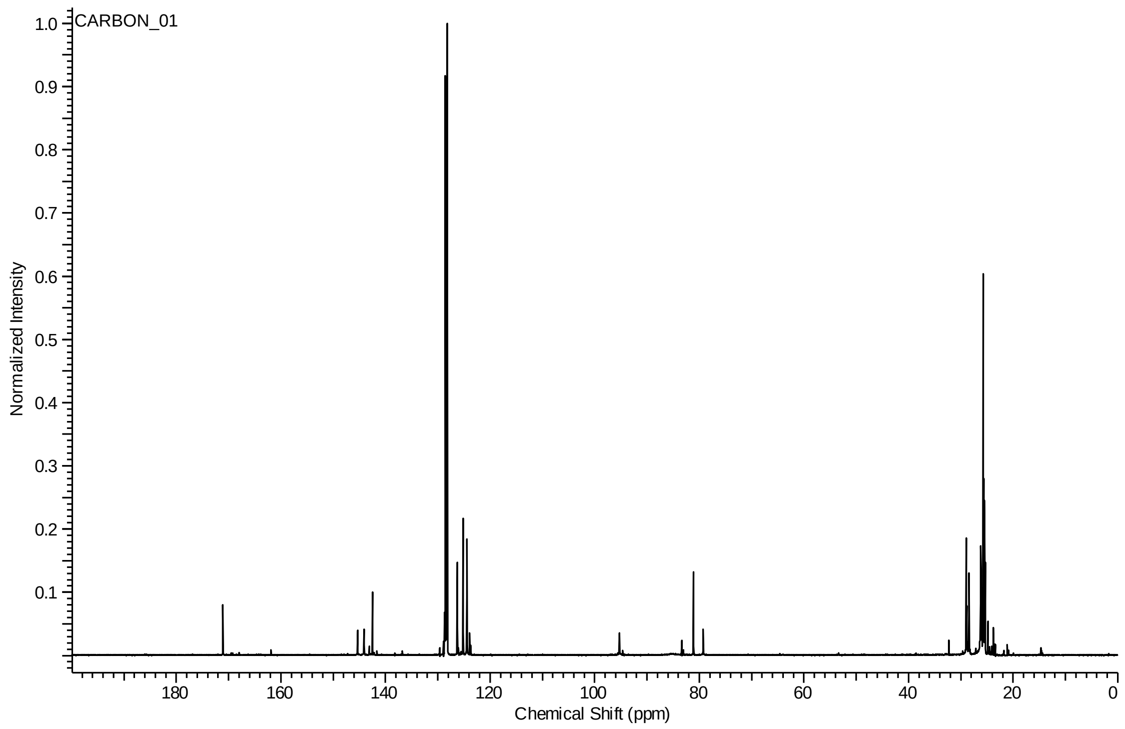

Supplementary Figure 8: $^{13}C\{^{1}H\}$ NMR spectrum of **10** in C_6D_6

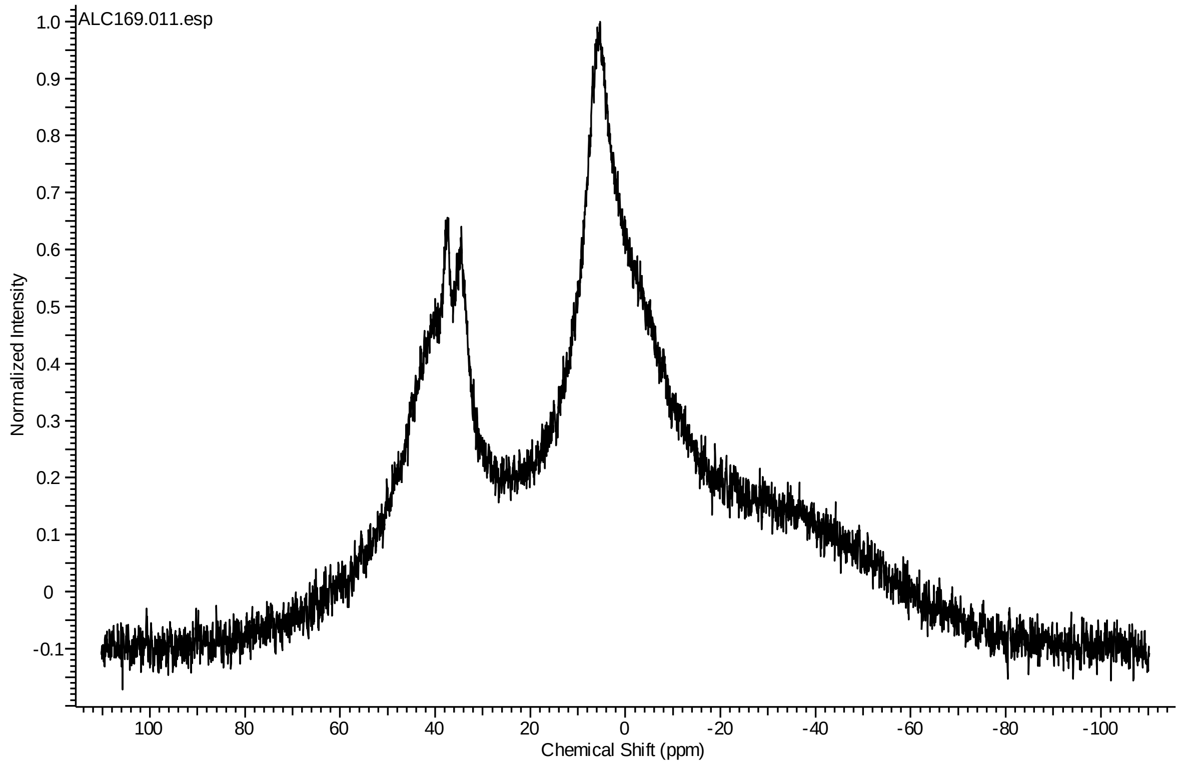

Supplementary Figure 9: $^{11}B\{^{1}H\}$ NMR spectrum of **10** in C_6D_6

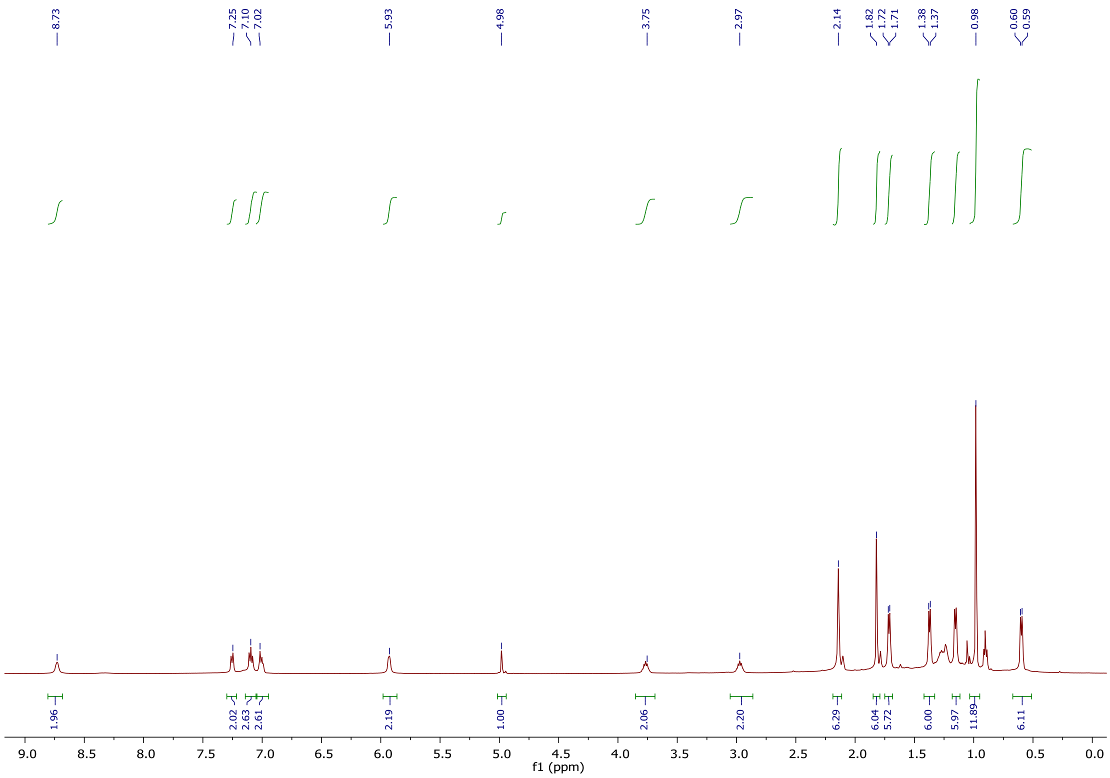

Supplementary Figure 10: ^{1}H NMR spectrum of **11** in d_8-Toluene

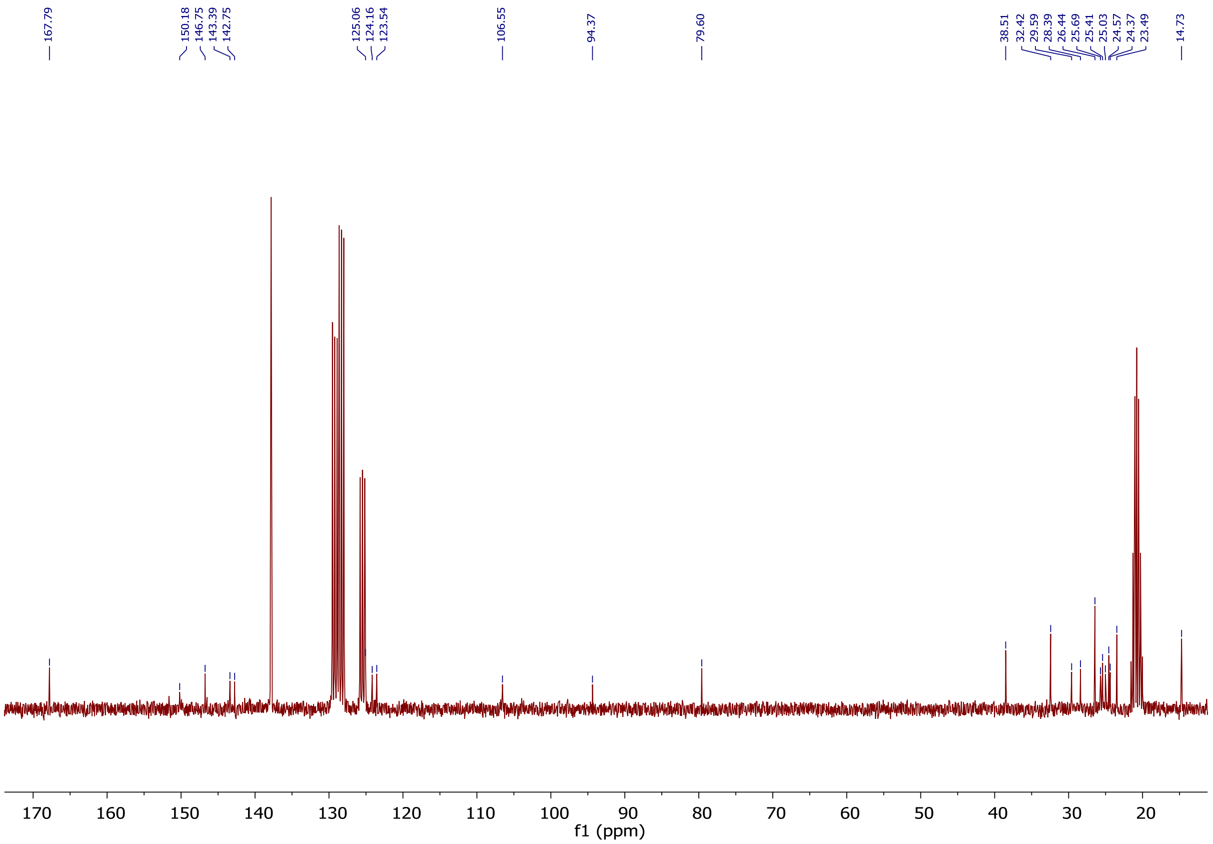

Supplementary Figure 11: ^{13}C{^{1}H} NMR spectrum of **11** in d_8-Toluene

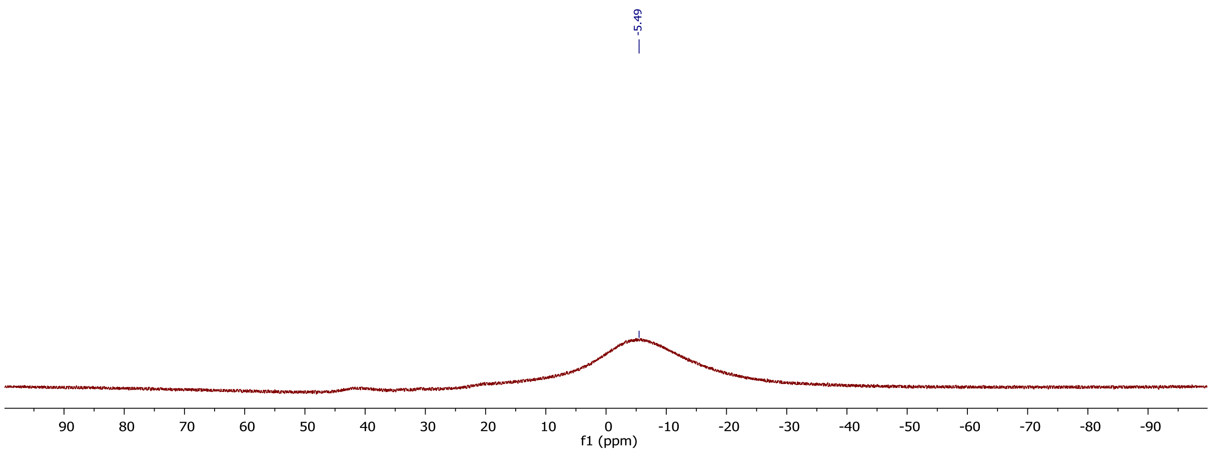

Supplementary Figure 12: $^{11}B\{^{1}H\}$ NMR spectrum of **11** in d_8-Toluene

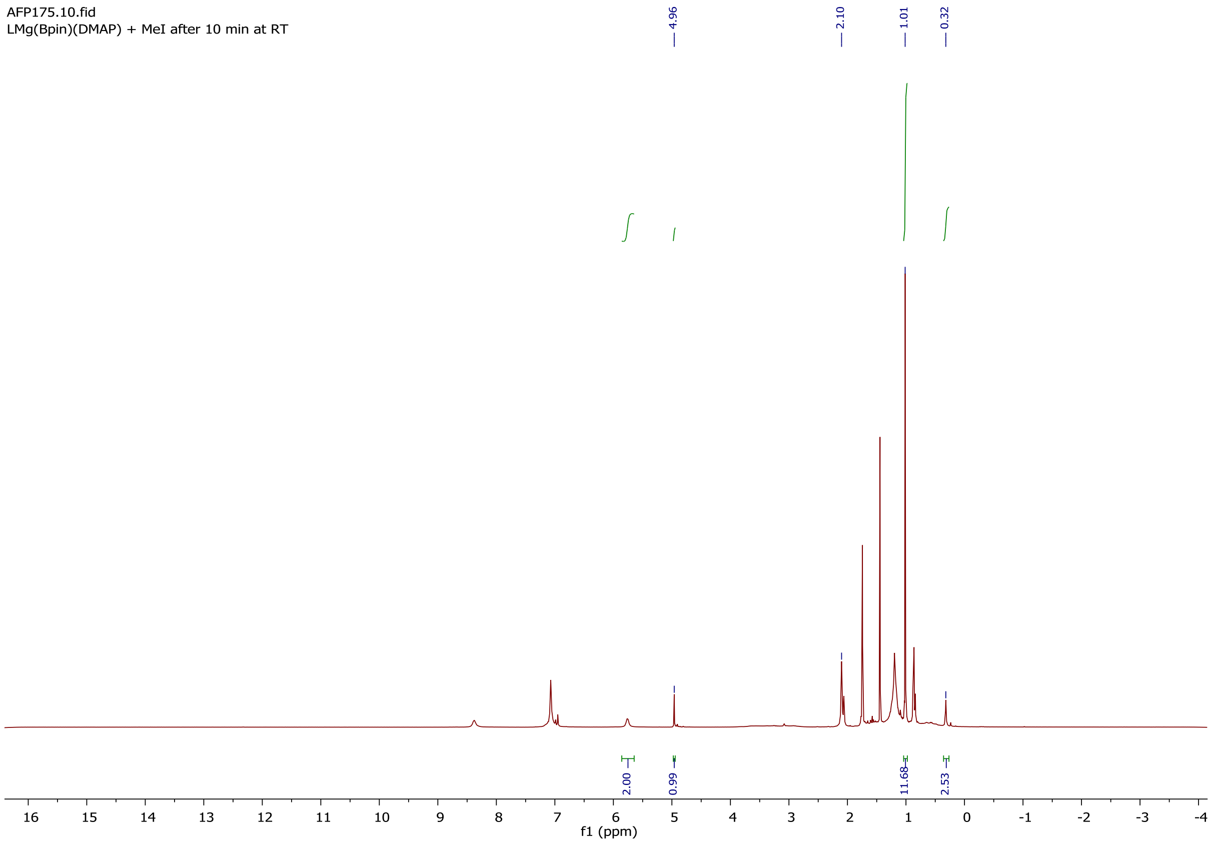

Supplementary Figure 13: ^{1}H NMR spectrum of the *in-situ* reaction between **11** and 1 equivalent of MeI in d_8-Toluene showing the formation of pinB-Me and **12**

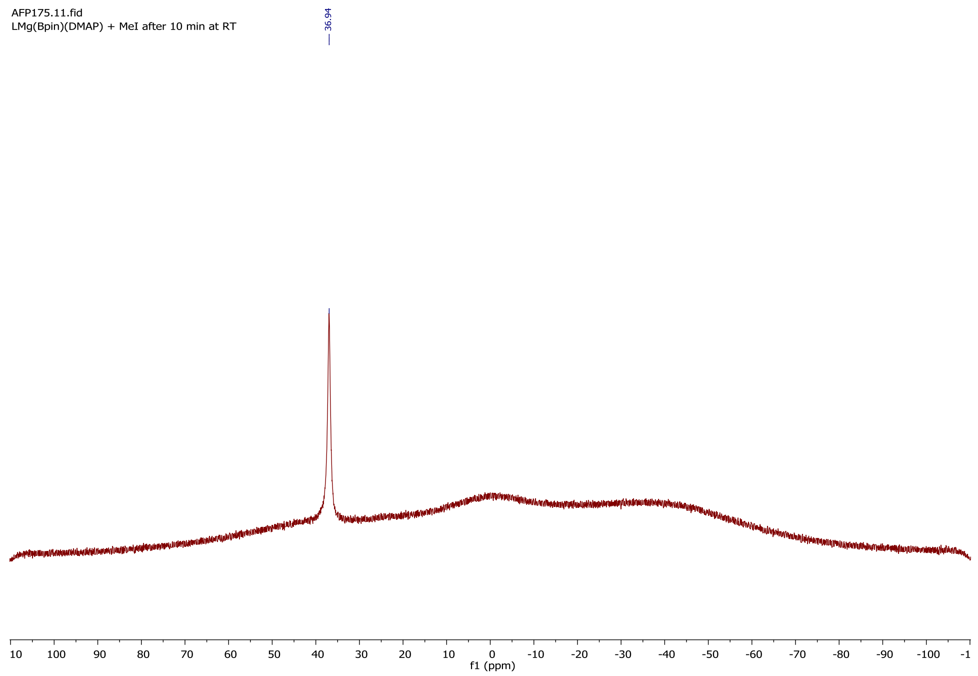

Supplementary Figure 14: $^{11}B\{^{1}H\}$ NMR spectrum of the *in-situ* reaction between **11** and 1 equivalent of MeI in d_8-toluene showing the formation of pinB-Me.

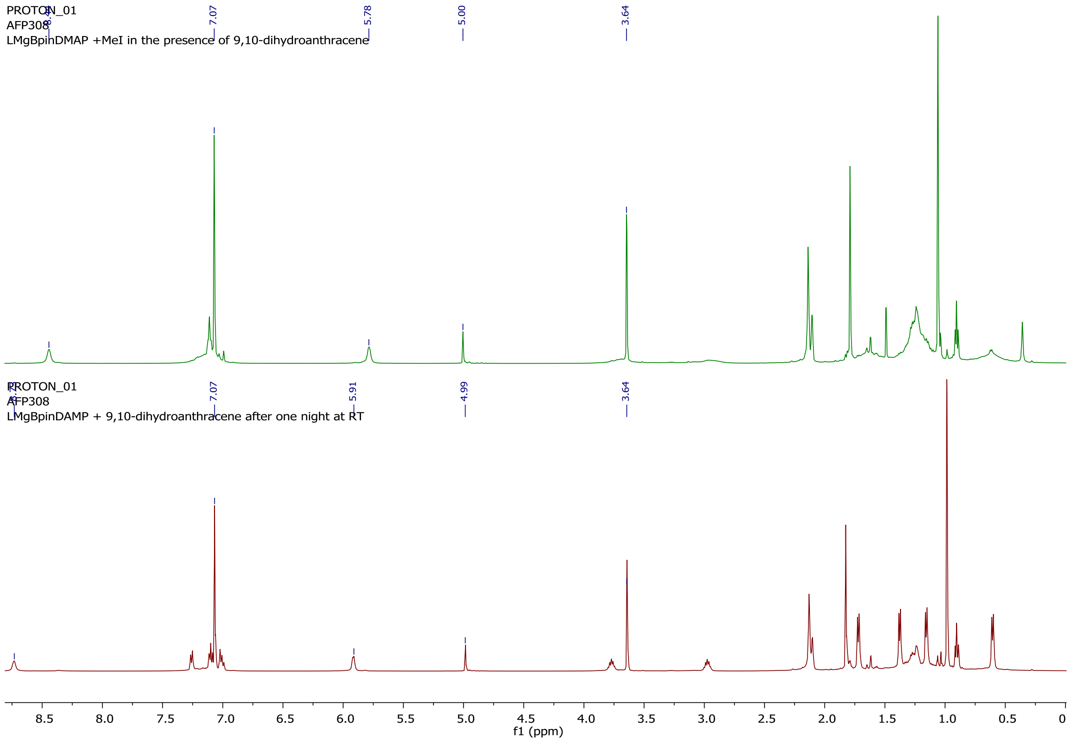

Supplementary Figure 15: ^{1}H NMR spectra of the *in-situ* reaction between **11** and 1 equivalent of MeI performed in the presence of an equimolar quantity of 9,10-dihydroanthracene (singlet resonance at δ 3.64 ppm) in d_8-toluene showing the formation of pinB-Me and **12**.

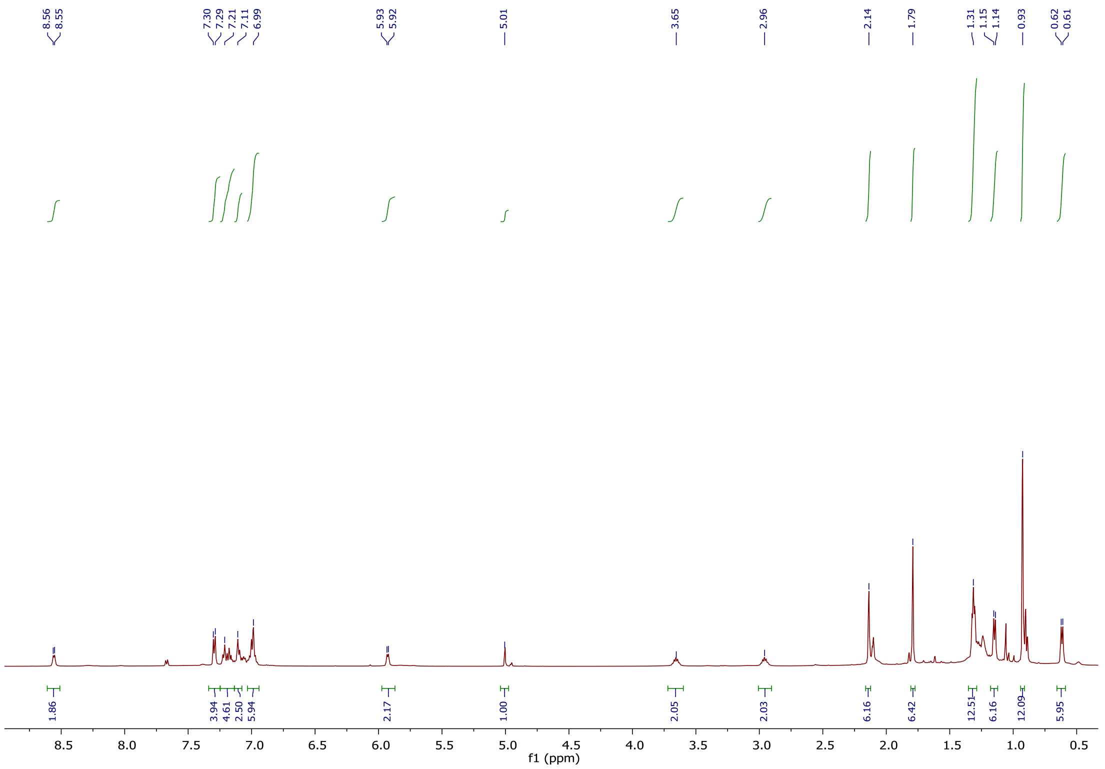

Supplementary Figure 16: ^{1}H NMR spectrum of **13** in d_8-Toluene

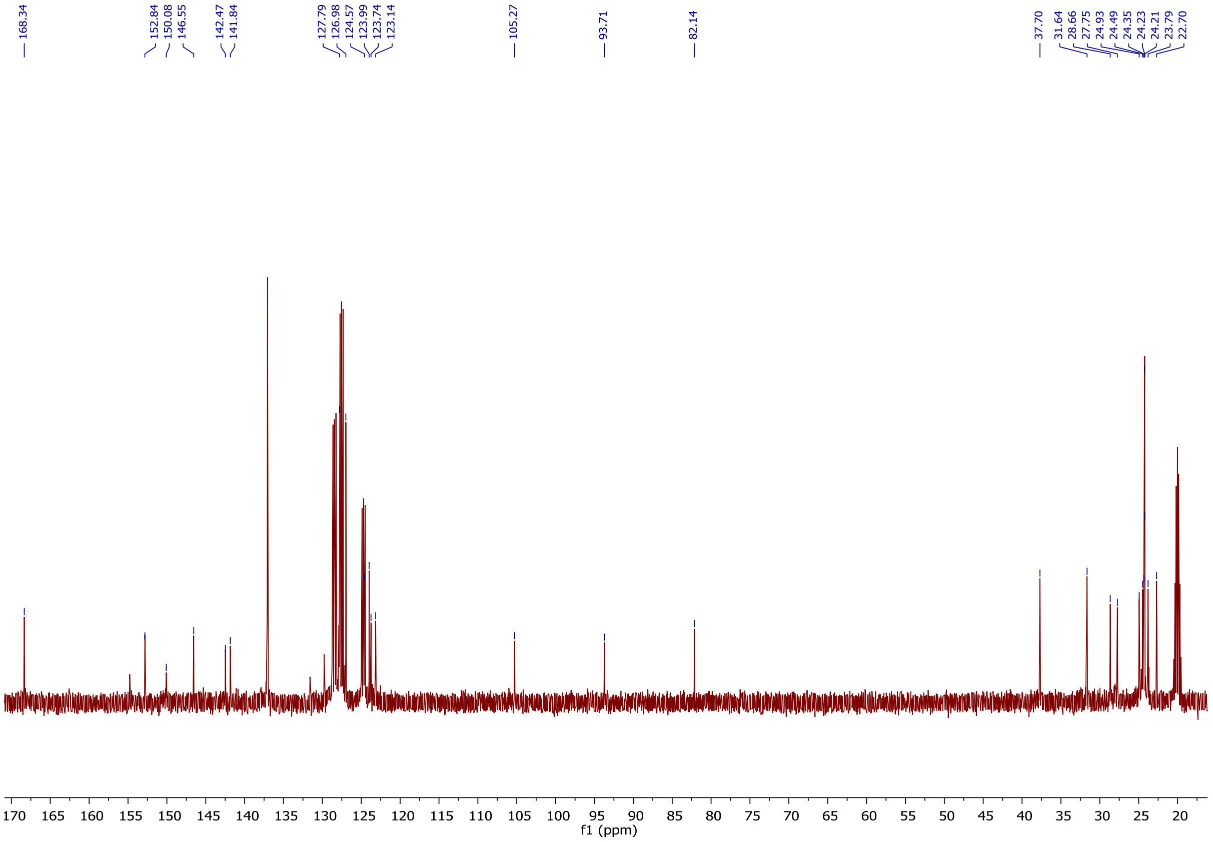

Supplementary Figure 17: ^{13}C{^{1}H} NMR spectrum of **13** in d_8-Toluene

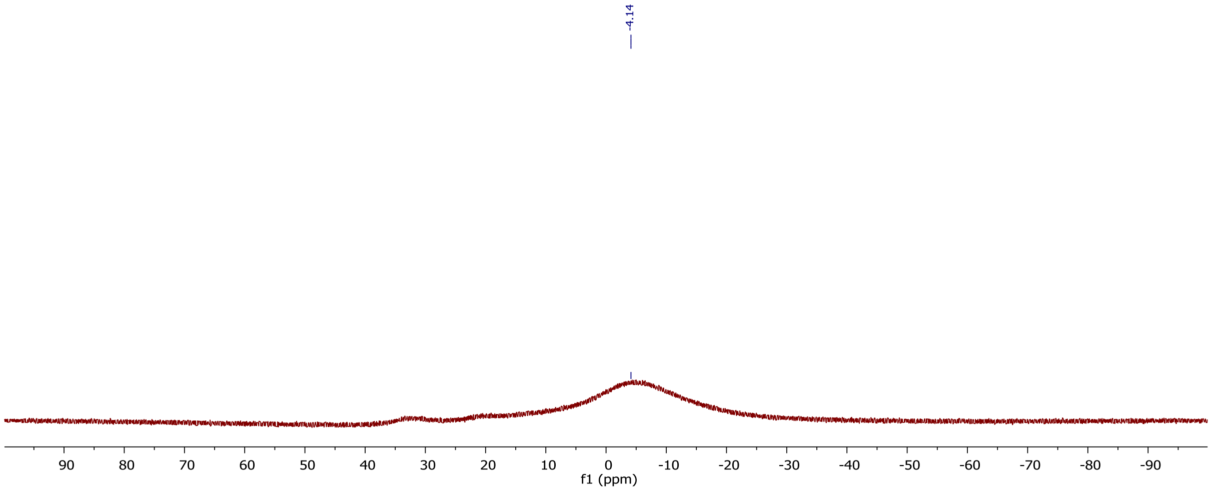

Supplementary Figure 18: $^{11}B\{^{1}H\}$ NMR spectrum of **13** in d_8-Toluene

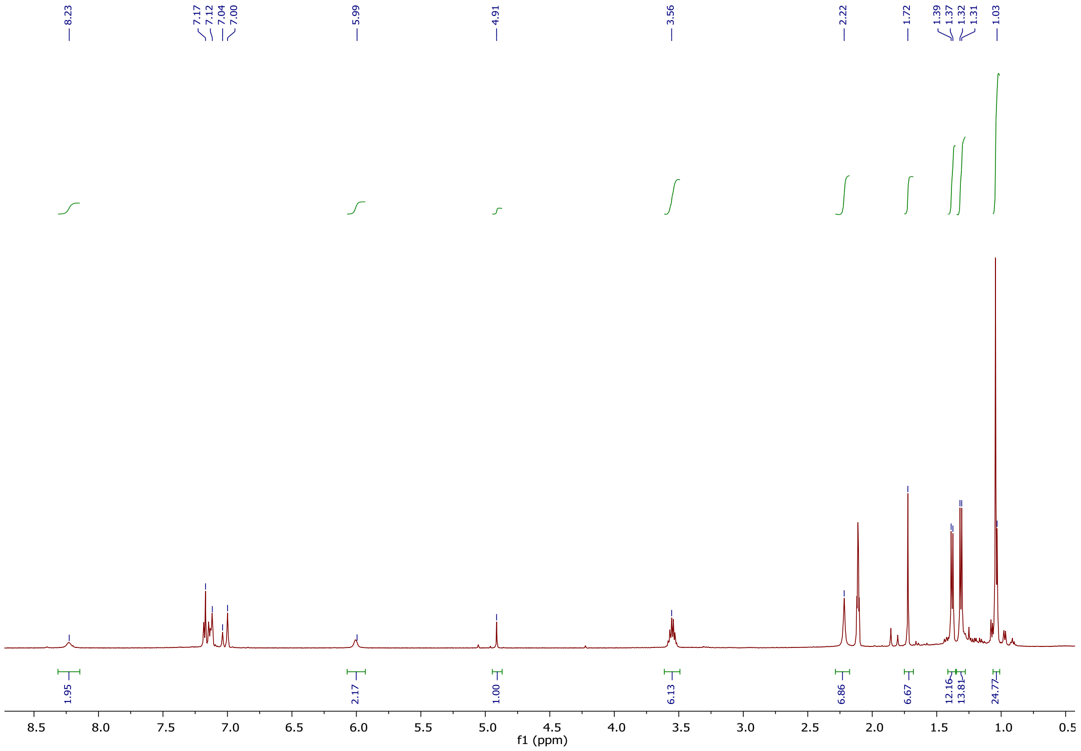

Supplementary Figure 19: ^{1}H NMR spectrum of **14** in d_8-Toluene

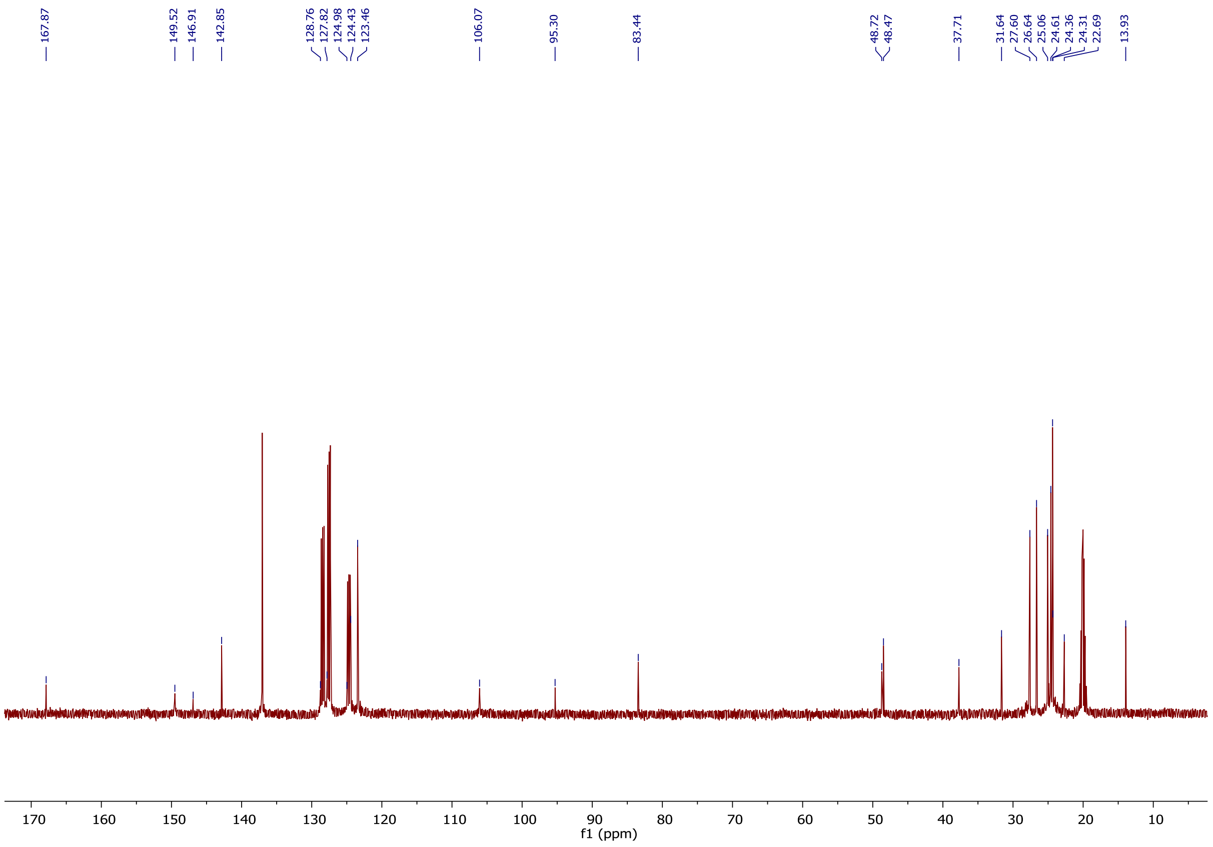

Supplementary Figure 20: $^{13}C\{^{1}H\}$ NMR spectrum of **14** in d_8-Toluene

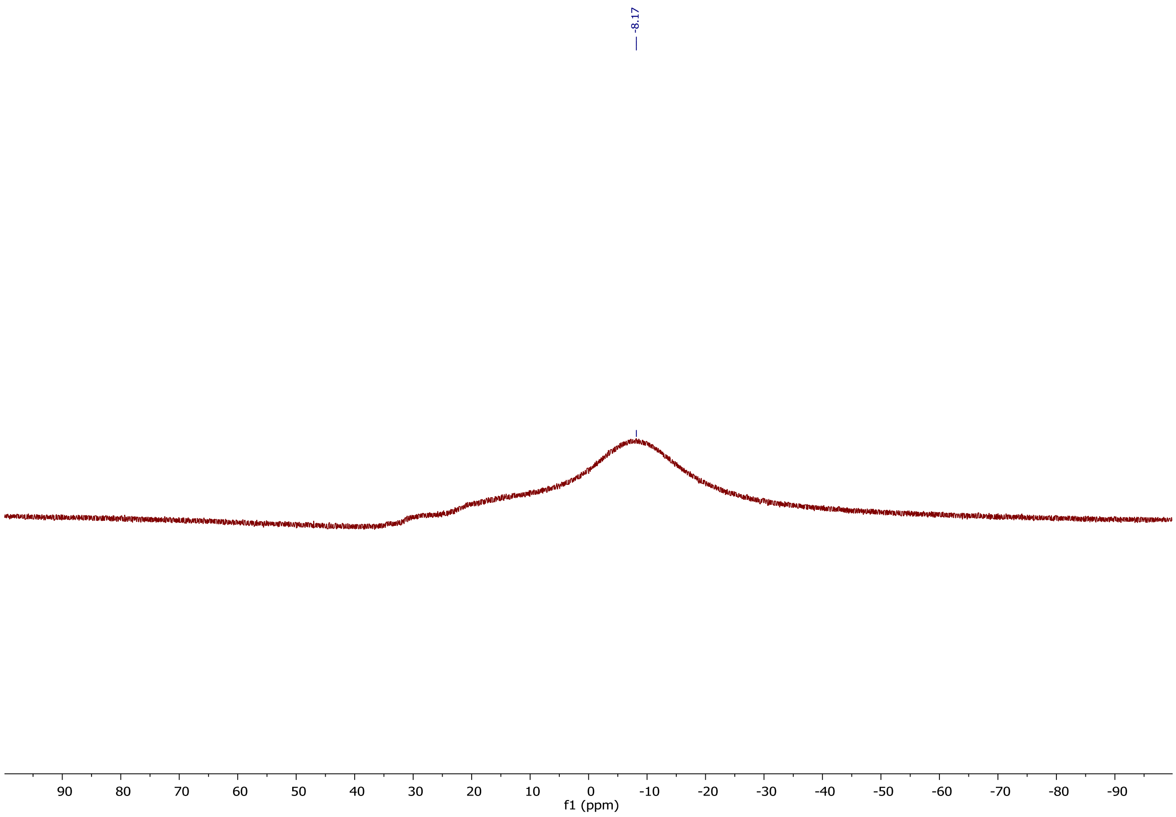

Supplementary Figure 21: $^{11}B\{^{1}H\}$ NMR spectrum of **14** in d_8-Toluene

Supplementary References

1. Dove, A. P., Gibson, V. C., Hormnirun, P., Marshall, E. L., Segal, J. A., White, A. J. P., Williams, D. J. Low coordinate magnesium chemistry supported by a bulky beta-diketiminate ligand. *Dalton. Trans.* 3088-3097 (2003).
2. Frisch, M. J.; Trucks, G. W.; Schlegel, H. B.; Scuseria, G. E.; Robb, M. A.; Cheeseman, J. R.; Scalmani, G.; Barone, V.; Mennucci, B.; Petersson, G. A.; Nakatsuji, H.; Caricato, M.; Li, X.; Hratchian, H. P.; Izmaylov, A. F.; Bloino, J.; Zheng, G.; Sonnenberg, J. L.; Hada, M.; Ehara, M.; Toyota, K.; Fukuda, R.; Hasegawa, J.; Ishida, M.; Nakajima, T.; Honda, Y.; Kitao, O.; Nakai, H.; Vreven, T.; Montgomery, J. A., Jr.; Peralta, J. E.; Ogliaro, F.; Bearpark, M.; Heyd, J. J.; Brothers, E.; Kudin, K. N.; Staroverov, V. N.; Kobayashi, R.; Normand, J.; Raghavachari, K.; Rendell, A.; Burant, J. C.; Iyengar, S. S.; Tomasi, J.; Cossi, M.; Rega, N.; Millam, J. M.; Klene, M.; Knox, J. E.; Cross, J. B.; Bakken, V.; Adamo, C.; Jaramillo, J.; Gomperts, R.; Stratmann, R. E.; Yazyev, O.; Austin, A. J.; Cammi, R.; Pomelli, C.; Ochterski, J. W.; Martin, R. L.; Morokuma, K.; Zakrzewski, V. G.; Voth, G. A.; Salvador, P.; Dannenberg, J. J.; Dapprich, S.; Daniels, A. D.; Farkas, O.; Foresman, J. B.; Ortiz, J. V.; Cioslowski, J.; Fox, D. J. Gaussian 09 (Revision D.01); Gaussian Inc.: Wallingford, CT (2009).
3. Andrae, D., Häußermann, U., Dolg, M., Stoll, H., Preuß, H. Energy-adjusted ab initio pseudopotentials for the 2nd and 3rd row transition elements. *Theor. Chim. Acta* **77**, 123−141 (1990).
4. Hariharan, P. C., Pople, J. A. The influence of polarization functions on molecular orbital hydrogenation energies. *Theor. Chim. Acta* **28**, 213−222 (1973).
5. Hehre, W. J., Ditchfield, R., Pople, J. A. Self-consistent molecular orbital methods 12. Further extensions of Gaussian-type basis sets for use in molecular orbital studies of organic molecules. *J. Chem. Phys.* **56**, 2257-2261 (1972).
6. Becke, A. D. Density functional exchange energy approximation with correct asymptotic behaviour. *Phys. Rev. A: At., Mol., Opt. Phys.* **38**, 3098-3100 (1988).
7. Perdew, J. P. Density functional approximation for the correlation energy of the inhomogeneous electron gas. *Phys. Rev. B: Condens. Matter Mater. Phys.* **33**, 8822−8824 (1986).
8. Tomasi, J., Mennucci, B., Cammi, R. Quantum mechanical continuum solvation models. *Chem. Rev.* **105**, 2999−3094 (2005).
9. Grimme, S., Ehrlich, S., L. Goerigk, L. Effect of the damping function in dispersion corrected density functional theory," *J. Comp. Chem.* **32**, 1456-1465 (2011).
10. Bourhis, L. J., Dolomanov, O. V., Gildea, R. J., Howard, J. A. K., Puschmann, H. The anatomy of a comprehensive constrained, restrained refinement program for the modern computing environment-Olex2 dissected. *Acta Cryst. A* **71**, 59-75 (2015).
11. Sheldrick, G. M. A short history of SHELX. *Acta Cryst. A* **64**, 112-122 (2008).
