# Supplementary material for: Easy access to nucleophilic boron through diborane to magnesium boryl metathesis
Source: Nat Commun. 2017 Apr 7;8:15022. doi: 10.1038/ncomms15022 (PMC5385571; doi:10.1038/ncomms15022)
Supplement: Supplementary Data 1 — Cartesian coordinates from DFT-calculated structures. [file ncomms15022-s2.docx]

**Cartesian Coordinates and Computed Energies (in Hartrees)**

**Bpin2**

SCF (BP86) Energy = -822.522371796

Enthalpy 0K = -822.169475

Enthalpy 298K = -822.148006

Free Energy 298K = -822.217576

Lowest Frequency = 12.9024 cm^-1^

Second Frequency = 38.1751 cm^-1^

SCF (Toluene) Energy = -822.526044458

SCF (BP86-D3BJ) Energy = -822.5928906

SCF (BS2) Energy = -822.737663787

B 0.85396 -0.00000 -0.00001

B -0.85395 -0.00001 -0.00001

O -1.61799 -1.05695 -0.46823

O -1.61798 1.05693 0.46822

C -3.01426 -0.60032 -0.51387

C -3.01424 0.60032 0.51387

C -3.91705 1.78002 0.13990

H -4.97620 1.47032 0.11227

H -3.81557 2.57770 0.89393

H -3.64870 2.20317 -0.83948

C -3.28160 0.16261 1.96504

H -3.05622 1.00443 2.63960

H -4.33429 -0.13049 2.11537

H -2.63667 -0.68448 2.25065

C -3.91708 -1.78000 -0.13989

H -4.97622 -1.47029 -0.11225

H -3.81561 -2.57769 -0.89391

H -3.64872 -2.20315 0.83950

C -3.28162 -0.16261 -1.96503

H -3.05625 -1.00442 -2.63960

H -4.33431 0.13050 -2.11536

H -2.63669 0.68448 -2.25065

O 1.61799 -1.05682 0.46849

O 1.61799 1.05682 -0.46851

C 3.01426 0.60016 -0.51404

C 3.01425 -0.60017 0.51404

C 3.28155 -0.16201 1.96508

H 3.05619 -1.00363 2.63990

H 4.33424 0.13117 2.11536

H 2.63660 0.68515 2.25042

C 3.91710 -1.77995 0.14045

H 4.97624 -1.47023 0.11275

H 3.81562 -2.57741 0.89471

H 3.64878 -2.20340 -0.83881

C 3.28158 0.16201 -1.96508

H 3.05622 1.00363 -2.63990

H 4.33427 -0.13117 -2.11534

H 2.63663 -0.68515 -2.25042

C 3.91709 1.77996 -0.14044

H 4.97624 1.47025 -0.11273

H 3.81561 2.57742 -0.89470

H 3.64876 2.20340 0.83883

**pinBBu**

SCF (BP86) Energy = -569.123611693

Enthalpy 0K = -568.827988

Enthalpy 298K = -568.811084

Free Energy 298K = -568.871244

Lowest Frequency = 17.1407 cm^-1^

Second Frequency = 53.5275 cm^-1^

SCF (Toluene) Energy = -569.125385795

SCF (BP86-D3BJ) Energy = -569.1741399

SCF (BS2) Energy = -569.267887458

O -0.86955 1.32355 -0.42098

O -0.18300 -0.73258 0.38809

C -2.09600 0.61210 -0.04689

C -2.54730 1.19499 1.30436

H -2.67048 2.28517 1.20025

H -3.50903 0.76697 1.63350

H -1.79541 1.01090 2.08938

C -3.15587 0.87903 -1.12021

H -4.08214 0.31845 -0.90581

H -3.40478 1.95284 -1.13647

H -2.79923 0.59839 -2.12243

C -1.60237 -0.88916 0.05313

C -2.26924 -1.72283 1.15171

H -3.35284 -1.82123 0.96660

H -1.83363 -2.73543 1.16496

H -2.12193 -1.27719 2.14676

C -1.65455 -1.63812 -1.29038

H -1.10344 -2.58741 -1.19226

H -2.69080 -1.86796 -1.58998

H -1.17991 -1.05036 -2.09324

B 0.19901 0.53546 -0.02082

C 1.69302 1.04254 -0.01374

H 1.83297 1.66855 0.89301

H 1.83638 1.74484 -0.85814

C 2.76818 -0.06372 -0.04099

H 2.59361 -0.76676 0.79568

H 2.65516 -0.66819 -0.96226

C 4.20767 0.47436 0.03493

H 4.32239 1.07303 0.96015

H 4.37762 1.17930 -0.80264

C 5.27346 -0.63192 0.00174

H 5.20596 -1.22365 -0.92865

H 6.29435 -0.21688 0.05981

H 5.14849 -1.33203 0.84698

**pinBMe**

SCF (BP86) Energy = -451.188548642

Enthalpy 0K = -450.976433

Enthalpy 298K = -450.963481

Free Energy 298K = -451.014202

Lowest Frequency = 11.9421 cm^-1^

Second Frequency = 90.9241 cm^-1^

SCF (Toluene) Energy = -451.190372527

SCF (BP86-D3BJ) Energy = -451.225917

SCF (BS2) Energy = -451.306903971

O -0.82032 -1.07695 -0.41263

O -0.81208 1.09464 0.38562

C 0.57062 -0.79236 -0.04709

C 0.81878 -1.48588 1.30450

H 0.58609 -2.55841 1.20421

H 1.86858 -1.38740 1.62801

H 0.16948 -1.06902 2.09198

C 1.48462 -1.38633 -1.12351

H 2.54239 -1.15035 -0.91470

H 1.37756 -2.48346 -1.13676

H 1.23158 -1.00868 -2.12541

C 0.58163 0.78822 0.04894

C 1.48265 1.36808 1.14391

H 2.54070 1.11590 0.95648

H 1.39221 2.46671 1.15524

H 1.20372 0.99455 2.14048

C 0.86780 1.47779 -1.29709

H 0.65007 2.55384 -1.20100

H 1.92221 1.36278 -1.59952

H 0.22741 1.07129 -2.09712

B -1.58021 0.01303 -0.01650

C -3.15422 0.01049 -0.00417

H -3.56405 -0.67848 -0.76055

H -3.52925 -0.33284 0.97764

H -3.56976 1.01690 -0.17336

**MeI**

SCF (BP86) Energy = -51.3700783605

Enthalpy 0K = -51.334246

Enthalpy 298K = -51.330110

Free Energy 298K = -51.358997

Lowest Frequency = 508.7578 cm^-1^

Second Frequency = 883.1508 cm^-1^

SCF (Toluene) Energy = -51.3715394765

SCF (BP86-D3BJ) Energy = -51.37473691

SCF (BS2) Energy = -335.769619801

C 0.00000 0.00000 -1.84855

H 0.00000 1.04641 -2.17483

H -0.90622 -0.52321 -2.17483

H 0.90622 -0.52321 -2.17483

I 0.00000 0.00000 0.33237

**benzophenone**

SCF (BP86) Energy = -576.623309768

Enthalpy 0K = -576.437366

Enthalpy 298K = -576.425337

Free Energy 298K = -576.475232

Lowest Frequency = 42.1834 cm^-1^

Second Frequency = 64.3203 cm^-1^

SCF (Toluene) Energy = -576.626357410

SCF (BP86-D3BJ) Energy = -576.6702729

SCF (BS2) Energy = -576.769376102

C 3.83941 -0.89879 -0.11835

C 2.71931 -1.55016 -0.65916

C 1.45644 -0.94021 -0.60625

C 1.30630 0.33687 -0.02143

C 2.44557 0.99589 0.49252

C 3.69992 0.37719 0.45782

H 4.82241 -1.38047 -0.15241

H 2.82915 -2.53389 -1.12745

H 0.58975 -1.44215 -1.04698

H 2.31530 1.99815 0.91192

H 4.57412 0.88947 0.87317

C -0.00001 1.08399 -0.00013

C -1.30631 0.33684 0.02136

C -1.45633 -0.94036 0.60596

C -2.44569 0.99594 -0.49225

C -2.71918 -1.55033 0.65899

H -0.58955 -1.44238 1.04642

C -3.70003 0.37723 -0.45742

H -2.31549 1.99826 -0.91151

C -3.83940 -0.89885 0.11854

H -2.82893 -2.53416 1.12709

H -4.57430 0.88960 -0.87250

H -4.82239 -1.38055 0.15270

O -0.00002 2.32332 -0.00022

**di-isopropyl carbodiimide**

SCF (BP86) Energy = -384.663567212

Enthalpy 0K = -384.465698

Enthalpy 298K = -384.453025

Free Energy 298K = -384.505161

Lowest Frequency = 13.7795 cm^-1^

Second Frequency = 22.0764 cm^-1^

SCF (Toluene) Energy = -384.6654055

SCF (BP86-D3BJ) Energy = -384.6924499

SCF (BS2) Energy = -384.7624458

C 0.08184 -0.42153 -0.50803

N -1.10697 -0.12093 -0.68341

N 1.24049 -0.85625 -0.50205

C -2.72455 1.39435 0.36238

H -3.52089 1.48813 1.12042

H -1.94257 2.13855 0.58552

H -3.15206 1.63284 -0.62625

C -3.22777 -1.08393 0.07032

H -2.80227 -2.10044 0.08673

H -4.03211 -1.02994 0.82396

H -3.66904 -0.91252 -0.92611

C 2.28775 1.42031 -0.23446

H 1.68455 1.68698 0.65157

H 3.25883 1.93777 -0.15366

H 1.76913 1.79904 -1.13087

C 3.21748 -0.65459 0.92549

H 4.21602 -0.19605 1.02486

H 2.64197 -0.43546 1.84153

H 3.33788 -1.74695 0.84905

C 2.49062 -0.10039 -0.31234

H 3.11264 -0.32864 -1.20056

C -2.14701 -0.03005 0.36222

H -1.70679 -0.23805 1.35863

**9**

SCF (BP86) Energy = -2220.57991976

Enthalpy 0K = -2219.487760

Enthalpy 298K = -2219.422702

Free Energy 298K = -2219.583100

Lowest Frequency = 18.9735 cm^-1^

Second Frequency = 26.5885 cm^-1^

SCF (Toluene) Energy = -2220.58694528

SCF (BP86-D3BJ) Energy = -2220.904161

SCF (BS2) Energy = -2420.36376068

Mg -0.34476 0.01378 -0.18714

O 1.54140 -2.22807 2.66404

O 0.23112 -0.49946 1.62808

O 2.35893 -3.05123 -0.58702

O 0.47307 -1.78737 -0.98025

N 0.51453 1.72437 -1.09139

N -2.31856 0.35138 -0.85786

C 0.52128 3.21091 -3.10961

H 1.23137 3.83727 -2.55179

H -0.27347 3.84981 -3.52516

H 1.06303 2.76611 -3.96307

C -0.07685 2.11136 -2.23709

C -1.29747 1.57224 -2.73842

H -1.55178 1.93263 -3.74029

C -2.37621 0.92362 -2.08493

C -3.68846 0.97689 -2.86537

H -4.44482 0.28346 -2.47490

H -3.49885 0.74795 -3.92734

H -4.11693 1.99339 -2.83081

C -3.58012 0.07622 -0.19956

C -4.27991 1.13606 0.45445

C -5.50795 0.85688 1.08681

H -6.04588 1.67243 1.58322

C -6.05569 -0.42852 1.09327

H -7.01385 -0.62228 1.58673

C -5.36109 -1.46468 0.46179

H -5.78584 -2.47393 0.46235

C -4.12896 -1.24233 -0.18402

C -3.45154 -2.41490 -0.89197

H -2.39781 -2.12895 -1.05143

C -4.08901 -2.67981 -2.27744

H -5.16444 -2.90677 -2.16873

H -3.61217 -3.54568 -2.76980

H -3.99583 -1.81357 -2.95154

C -3.47321 -3.71728 -0.06284

H -4.49158 -4.13664 0.01548

H -3.09036 -3.56385 0.95815

H -2.84912 -4.48910 -0.54735

C -3.75936 2.57485 0.51098

H -2.77726 2.60376 0.00859

C -4.69983 3.55854 -0.22462

H -5.68564 3.61324 0.27025

H -4.87252 3.26009 -1.27138

H -4.27065 4.57572 -0.22645

C -3.56264 3.03956 1.97213

H -4.51365 3.02218 2.53234

H -3.18275 4.07550 2.00169

H -2.84709 2.39704 2.51180

C 1.66127 2.46199 -0.59421

C 1.46942 3.35241 0.50686

C 2.58182 4.02219 1.04927

H 2.43604 4.70200 1.89370

C 3.86843 3.84311 0.53151

H 4.72197 4.36877 0.97182

C 4.04690 2.99050 -0.55883

H 5.05064 2.85487 -0.97631

C 2.96910 2.29261 -1.14167

C 0.07563 3.66519 1.05162

H -0.55049 2.76348 0.91397

C -0.57072 4.80491 0.22652

H 0.04535 5.71953 0.28797

H -1.57765 5.04808 0.60918

H -0.66802 4.53353 -0.83720

C 0.06676 4.02383 2.55137

H 0.61356 3.27809 3.15151

H -0.96954 4.07474 2.92381

H 0.52274 5.01173 2.74049

C 3.28221 1.39866 -2.34317

H 2.32990 0.97938 -2.70761

C 4.20878 0.22372 -1.95175

H 4.44417 -0.39762 -2.83533

H 3.76039 -0.42235 -1.18001

H 5.16769 0.59505 -1.55095

C 3.92666 2.19470 -3.50432

H 3.32408 3.07013 -3.79544

H 4.04744 1.54914 -4.39225

H 4.93023 2.56185 -3.22754

C 0.71923 -2.53986 -2.25600

C 1.30645 -1.54898 -3.26490

H 1.45104 -2.03087 -4.24660

H 2.27400 -1.15597 -2.92583

H 0.60746 -0.70550 -3.40091

C -0.60019 -3.08965 -2.79294

H -1.26580 -2.26321 -3.08956

H -1.12198 -3.71574 -2.05601

H -0.40765 -3.70185 -3.69148

C 1.73639 -3.64723 -1.76936

C 2.84369 -3.97658 -2.77714

H 3.44517 -3.08997 -3.02619

H 2.42159 -4.39293 -3.70824

H 3.51793 -4.73147 -2.34178

C 1.05731 -4.94472 -1.29309

H 0.62269 -5.51401 -2.13197

H 0.26546 -4.73848 -0.55488

H 1.81513 -5.57543 -0.80189

C -0.37602 -0.86951 2.90607

C -1.90023 -0.81125 2.79138

H -2.24597 0.22419 2.62952

H -2.37059 -1.17054 3.72328

H -2.28208 -1.42729 1.96252

C 0.10443 0.12876 3.97487

H 1.19142 0.04849 4.12432

H -0.40125 -0.04188 4.94089

H -0.12774 1.15469 3.64460

C 0.20857 -2.32100 3.14062

C 0.25527 -2.73479 4.62236

H 0.66183 -3.75681 4.70278

H -0.75003 -2.73021 5.08018

H 0.91376 -2.06703 5.19770

C -0.55993 -3.40135 2.34292

H -0.67724 -3.10688 1.28621

H -1.56111 -3.60756 2.76108

H 0.02589 -4.33463 2.37468

B 1.63895 -1.29504 1.51821

B 1.53973 -2.08466 -0.04812

C 2.92915 -0.32134 1.71530

H 3.02134 0.42936 0.90451

H 2.79079 0.27020 2.64468

C 4.25417 -1.10467 1.82956

H 4.15187 -1.86253 2.63028

H 4.43212 -1.68159 0.89849

C 5.48588 -0.22655 2.11659

H 5.31879 0.33207 3.05884

H 5.57591 0.54480 1.32605

C 6.79762 -1.02175 2.21680

H 7.66256 -0.36830 2.42856

H 6.74423 -1.77660 3.02190

H 7.00958 -1.56239 1.27647

**10**

SCF (BP86) Energy = -2473.98441885

Enthalpy 0K = -2472.834819

Enthalpy 298K = -2472.765137

Free Energy 298K = -2472.935016

Lowest Frequency = 17.5079 cm^-1^

Second Frequency = 21.5821 cm^-1^

SCF (Toluene) Energy = -2473.99078163

SCF (BP86-D3BJ) Energy = -2474.334868

SCF (BS2) Energy = -2673.83452119

Mg 0.67877 0.09794 0.30557

O -0.80695 -0.49136 1.71063

O -2.47834 -2.07516 1.77441

O -0.62630 -0.05197 -1.16038

O -1.54761 -2.15272 -1.70192

O -4.21028 -0.38213 -2.12504

N 2.39773 -1.07878 0.58192

N 1.57685 1.89721 0.85331

C 2.68345 2.95345 2.82395

H 3.60813 3.44554 2.47761

H 2.85158 2.63921 3.86606

H 1.88280 3.70860 2.79507

C 2.34411 1.74783 1.95638

C 2.90138 0.51135 2.38209

H 3.44144 0.56998 3.33182

C 3.06976 -0.72818 1.69782

C 4.04562 -1.69572 2.35991

H 3.51216 -2.27231 3.13818

H 4.86895 -1.15828 2.85520

H 4.46280 -2.41762 1.64432

C 1.38776 3.22661 0.32305

C 0.11885 3.86975 0.42174

C -0.05173 5.14693 -0.14751

H -1.02854 5.63651 -0.06508

C 0.99437 5.80354 -0.80119

H 0.84322 6.79777 -1.23462

C 2.23765 5.17063 -0.89345

H 3.06096 5.67803 -1.40888

C 2.46279 3.89231 -0.34652

C -1.06744 3.24304 1.14969

H -0.76148 2.24018 1.49448

C -1.45231 4.06513 2.40151

H -0.61259 4.14451 3.11328

H -2.30492 3.60150 2.92822

H -1.75252 5.09127 2.12572

C -2.28152 3.06807 0.21307

H -2.67673 4.04556 -0.11721

H -3.09266 2.52061 0.71992

H -2.00510 2.48787 -0.68195

C 3.84923 3.27180 -0.54164

H 3.87440 2.30140 -0.01715

C 4.97930 4.15625 0.03683

H 5.06894 5.10740 -0.51684

H 5.95077 3.63746 -0.03916

H 4.81167 4.41001 1.09684

C 4.11950 3.00583 -2.04010

H 3.37392 2.31791 -2.47131

H 5.12070 2.56225 -2.18078

H 4.08587 3.94348 -2.62209

C 2.84260 -2.23994 -0.16388

C 2.23763 -3.51740 0.02694

C 2.71440 -4.61532 -0.71682

H 2.25193 -5.59638 -0.56329

C 3.75791 -4.48113 -1.63551

H 4.11811 -5.34890 -2.19808

C 3.88623 -2.08523 -1.13020

C 4.32805 -3.22024 -1.83844

H 5.13507 -3.11017 -2.56960

C 1.11068 -3.76323 1.02781

H 0.82373 -2.77977 1.43721

C 1.59241 -4.64959 2.20128

H 1.87240 -5.65547 1.84157

H 0.78805 -4.78183 2.94737

H 2.47189 -4.22527 2.71400

C -0.14226 -4.38320 0.36937

H -0.53299 -3.75344 -0.44861

H -0.94477 -4.49517 1.11864

H 0.07591 -5.38805 -0.03501

C 4.55044 -0.73412 -1.41300

H 3.84661 0.05205 -1.08317

C 5.85964 -0.55799 -0.60700

H 5.68463 -0.59810 0.47951

H 6.32966 0.41468 -0.83764

H 6.58378 -1.35202 -0.86303

C 4.83442 -0.52330 -2.91699

H 5.63115 -1.19456 -3.28245

H 5.17671 0.50835 -3.09940

H 3.93666 -0.70061 -3.53153

C -0.43161 -0.18584 -2.60451

C -0.93472 -1.68971 -2.91352

C -1.25654 0.91404 -3.29668

H -2.32582 0.82413 -3.05909

H -1.14149 0.86710 -4.39274

H -0.89975 1.90236 -2.95901

C 1.05486 0.06082 -2.89696

H 1.32941 1.08807 -2.59454

H 1.26527 -0.02868 -3.97619

H 1.70755 -0.65476 -2.37056

C 0.21945 -2.66549 -3.22424

H 0.96796 -2.69522 -2.41734

H 0.72802 -2.42056 -4.17349

H -0.20257 -3.67958 -3.31855

C -1.95473 -1.76399 -4.07160

H -2.24925 -2.81830 -4.20677

H -1.52353 -1.41045 -5.02589

H -2.86378 -1.18907 -3.84525

C -1.06656 -0.70342 3.17444

C -2.40116 -1.57008 3.14558

C 0.14103 -1.42424 3.78185

H 0.29545 -2.42146 3.34711

H 0.00575 -1.54199 4.87038

H 1.05027 -0.82251 3.61339

C -1.21231 0.67317 3.82680

H -0.26389 1.22873 3.74117

H -1.44268 0.56443 4.90078

H -2.00889 1.26852 3.35960

C -2.39227 -2.77879 4.09084

H -3.32727 -3.34565 3.95541

H -2.33708 -2.45946 5.14583

H -1.55278 -3.45911 3.88435

C -3.68104 -0.74492 3.37258

H -3.77887 0.05057 2.61781

H -3.71435 -0.30822 4.38498

H -4.55003 -1.41316 3.25927

B -1.69356 -1.31971 0.93456

B -1.81547 -1.03486 -0.77740

B -3.43128 -0.33057 -0.96218

O -4.18306 0.21860 0.09210

C -5.46886 0.33050 -1.89574

C -5.29418 1.75002 -2.46525

H -5.01449 1.67734 -3.52907

H -6.22570 2.33724 -2.39406

H -4.49359 2.29470 -1.93869

C -6.58240 -0.40483 -2.64996

H -7.56507 0.06036 -2.45685

H -6.39076 -0.35435 -3.73456

H -6.63264 -1.46608 -2.36491

C -5.58754 0.29777 -0.32427

C -6.22230 1.54181 0.30601

H -7.26774 1.66136 -0.02862

H -6.23103 1.44101 1.40429

H -5.66767 2.45742 0.05143

C -6.27857 -0.97348 0.20263

H -6.15325 -1.02391 1.29615

H -7.35888 -0.97423 -0.02193

H -5.82548 -1.87912 -0.23156

**11**

SCF (BP86) Energy = -2033.73527839

Enthalpy 0K = -2032.782167

Enthalpy 298K = -2032.722299

Free Energy 298K = -2032.879997

Lowest Frequency = 6.8807 cm^-1^

Second Frequency = 11.4325 cm^-1^

SCF (Toluene) Energy = -2033.74238983

SCF (BP86-D3BJ) Energy = -2034.004051

SCF (BS2) Energy = -2233.47329677

Mg 0.15023 -0.01532 -0.11615

O 2.27725 -0.08384 2.48866

O 0.09814 0.14180 3.18101

N 0.35464 -1.59923 -1.51504

N 0.53864 1.46365 -1.58736

N -2.05316 0.11106 0.07780

N -6.25270 0.37862 0.54207

C 0.18742 -2.55506 -3.80102

H 1.16551 -3.06663 -3.79749

H -0.02833 -2.22806 -4.82885

H -0.55552 -3.31294 -3.50315

C 0.20025 -1.37754 -2.83345

C 0.08411 -0.08965 -3.42586

H -0.08001 -0.10520 -4.50636

C 0.35080 1.20196 -2.89385

C 0.47103 2.32722 -3.91465

H -0.12086 3.20742 -3.61512

H 0.14699 1.99872 -4.91333

H 1.51785 2.67031 -3.98793

C 0.63809 -2.93939 -1.06496

C 1.99478 -3.35091 -0.90016

C 3.17449 -2.42902 -1.21542

H 2.76031 -1.45159 -1.51871

C 4.05893 -2.20043 0.03128

H 3.48995 -1.73169 0.85092

H 4.90652 -1.53785 -0.21714

H 4.48230 -3.15099 0.40255

C 4.02069 -2.96716 -2.39257

H 4.84102 -2.26759 -2.63161

H 3.41572 -3.10336 -3.30533

H 4.47545 -3.94323 -2.14594

C 2.26012 -4.65125 -0.42848

H 3.30218 -4.96941 -0.30867

C 1.22863 -5.54085 -0.10968

H 1.45731 -6.54862 0.25351

C -0.09879 -5.12330 -0.25245

H -0.91144 -5.81143 0.00831

C -0.41931 -3.83503 -0.72321

C -1.89220 -3.43524 -0.81830

H -1.93252 -2.42171 -1.25154

C -2.70914 -4.37505 -1.73384

H -2.73711 -5.40468 -1.33544

H -2.28958 -4.42653 -2.75297

H -3.75312 -4.02329 -1.81473

C -2.53212 -3.37151 0.58750

H -3.57126 -3.00184 0.52896

H -1.96584 -2.70029 1.25331

H -2.55411 -4.37057 1.05848

C 1.00207 2.77292 -1.20164

C 2.40435 3.02430 -1.11729

C 3.44793 1.95442 -1.44398

H 2.90596 1.04416 -1.75405

C 4.28750 1.60027 -0.19522

H 5.03982 0.83078 -0.44313

H 3.65593 1.21105 0.62069

H 4.83124 2.48522 0.18186

C 4.36438 2.37798 -2.61479

H 4.96047 3.27179 -2.35790

H 3.78643 2.61490 -3.52458

H 5.07173 1.56757 -2.86418

C 2.84491 4.29552 -0.70113

H 3.92200 4.48942 -0.63864

C 1.94262 5.31042 -0.36425

H 2.30665 6.29318 -0.04568

C 0.56962 5.05104 -0.43139

H -0.14231 5.83887 -0.15832

C 0.07642 3.79714 -0.84206

C -1.43567 3.57299 -0.86375

H -1.61150 2.55202 -1.24197

C -2.16811 4.55792 -1.80294

H -3.25023 4.33723 -1.82668

H -1.78791 4.49973 -2.83710

H -2.05015 5.60271 -1.46498

C -2.02299 3.65381 0.56378

H -1.51785 2.94826 1.24360

H -3.10109 3.41399 0.55806

H -1.90457 4.66814 0.98521

C -2.89073 0.13850 -0.99165

H -2.40773 0.08837 -1.97568

C -4.27632 0.22506 -0.89436

H -4.86995 0.24180 -1.81029

C -4.88936 0.29133 0.39161

C -4.00662 0.26197 1.50929

H -4.38330 0.30798 2.53278

C -2.63154 0.17318 1.30637

H -1.93700 0.14961 2.15653

C -7.11906 0.40614 -0.63372

H -6.90517 1.27850 -1.28015

H -8.16614 0.47391 -0.30766

H -7.00997 -0.51068 -1.24358

C -6.83675 0.44965 1.87940

H -6.59538 -0.44846 2.47886

H -7.92997 0.51612 1.79155

H -6.48277 1.33979 2.43314

B 0.94453 0.01560 2.05953

C 2.34833 0.19273 3.93070

C 0.87574 -0.09912 4.40598

C 3.42260 -0.71237 4.54386

H 4.40693 -0.45918 4.11648

H 3.48141 -0.57254 5.63799

H 3.22690 -1.77485 4.33566

C 2.75772 1.67011 4.07676

H 3.70722 1.83187 3.54130

H 2.00215 2.33720 3.63149

H 2.90400 1.95422 5.13344

C 0.34881 0.82952 5.50556

H 0.93881 0.72015 6.43285

H 0.37762 1.88464 5.19488

H -0.69787 0.57198 5.74022

C 0.63819 -1.56950 4.79771

H -0.44480 -1.73890 4.91682

H 1.00420 -2.25156 4.01356

H 1.13342 -1.82689 5.75005

**12**

SCF (BP86) Energy = -1634.03135960

Enthalpy 0K = -1633.253032

Enthalpy 298K = -1633.202284

Free Energy 298K = -1633.342775

Lowest Frequency = 3.3210 cm^-1^

Second Frequency = 13.0599 cm^-1^

SCF (Toluene) Energy = -1634.04156257

SCF (BP86-D3BJ) Energy = -1634.267397

SCF (BS2) Energy = -2118.052155

Mg 0.18584 0.00002 -0.28585

N 0.76110 -1.54108 0.99311

N 0.75726 1.54281 0.99283

N -1.98464 -0.00251 -0.21953

N -6.20681 -0.00732 0.06609

C 1.02726 -2.44264 3.29151

H 0.33795 -3.27807 3.08807

H 0.89802 -2.11346 4.33299

H 2.04663 -2.85161 3.18274

C 0.80970 -1.29272 2.31711

C 0.70753 0.00096 2.89959

H 0.71808 0.00107 3.99230

C 0.80652 1.29480 2.31687

C 1.02125 2.44543 3.29107

H 2.03891 2.85822 3.18081

H 0.89467 2.11574 4.33270

H 0.32857 3.27832 3.08867

C 1.06772 2.87735 0.53090

C 2.41810 3.21563 0.22209

C 2.70226 4.51358 -0.24494

H 3.73947 4.77695 -0.48129

C 1.69383 5.46709 -0.41953

H 1.93732 6.47087 -0.78363

C 0.36926 5.11920 -0.13555

H -0.42647 5.85830 -0.28424

C 0.03070 3.83619 0.33668

C -1.44114 3.51502 0.59817

H -1.48969 2.48767 0.99695

C -2.07747 4.45605 1.64625

H -1.53588 4.42885 2.60717

H -3.12573 4.16644 1.83971

H -2.08225 5.50400 1.29834

C -2.25235 3.54958 -0.71756

H -2.27156 4.56790 -1.14473

H -3.29729 3.23796 -0.54146

H -1.81460 2.88062 -1.47667

C 3.56812 2.21554 0.35736

H 3.14082 1.26067 0.71043

C 4.23803 1.95531 -1.01113

H 3.50911 1.57761 -1.74725

H 5.04572 1.20945 -0.91020

H 4.68698 2.87817 -1.41924

C 4.61639 2.67429 1.39707

H 5.09609 3.62144 1.09268

H 5.41284 1.91688 1.50260

H 4.16800 2.83395 2.39268

C 1.07520 -2.87482 0.53135

C 2.42666 -3.20992 0.22380

C 2.71424 -4.50704 -0.24343

H 3.75227 -4.76794 -0.47892

C 1.70817 -5.46282 -0.41929

H 1.95432 -6.46590 -0.78353

C 0.38255 -5.11811 -0.13635

H -0.41133 -5.85902 -0.28591

C 0.04060 -3.83603 0.33598

C -1.43217 -3.51846 0.59664

H -1.48343 -2.49122 0.99534

C -2.24272 -3.55510 -0.71942

H -1.80630 -2.88510 -1.47839

H -3.28850 -3.24604 -0.54376

H -2.25929 -4.57348 -1.14658

C -2.06657 -4.46097 1.64458

H -2.06843 -5.50898 1.29685

H -3.11567 -4.17405 1.83749

H -1.52549 -4.43216 2.60574

C 3.57423 -2.20720 0.36038

H 3.14462 -1.25378 0.71455

C 4.62363 -2.66460 1.39952

H 4.17560 -2.82689 2.39487

H 5.41803 -1.90520 1.50617

H 5.10593 -3.61002 1.09387

C 4.24352 -1.94376 -1.00783

H 4.69451 -2.86514 -1.41701

H 5.04953 -1.19620 -0.90601

H 3.51376 -1.56689 -1.74355

C -2.77566 -0.00334 -1.32501

H -2.24028 -0.00269 -2.28254

C -4.16564 -0.00484 -1.28324

H -4.71683 -0.00527 -2.22508

C -4.83820 -0.00567 -0.02649

C -4.00362 -0.00469 1.13105

H -4.42518 -0.00508 2.13772

C -2.62136 -0.00319 0.98346

H -1.97287 -0.00248 1.86867

C -6.85324 -0.00690 1.37720

H -6.58127 -0.90268 1.96662

H -7.94322 -0.00856 1.24047

H -6.58372 0.89069 1.96502

C -7.02212 -0.00762 -1.14795

H -6.83291 0.88993 -1.76602

H -8.08425 -0.01020 -0.86747

H -6.82914 -0.90310 -1.76778

I 0.74427 0.00042 -2.94120

**13**

SCF (BP86) Energy = -2610.41729271

Enthalpy 0K = -2609.276317

Enthalpy 298K = -2609.204318

Free Energy 298K = -2609.387627

Lowest Frequency = 10.0702 cm^-1^

Second Frequency = 19.5868 cm^-1^

SCF (Toluene) Energy = -2610.42623951

SCF (BP86-D3BJ) Energy = -2610.775838

SCF (BS2) Energy = -2810.29764111

Mg 0.73103 -0.49060 0.35756

O -0.45411 0.62176 -0.54816

O -3.37364 0.68552 -0.54707

O -3.34723 2.95084 -0.99930

N 2.35167 -1.58277 -0.44802

N 1.73603 -0.09962 2.16397

N -0.61390 -2.12579 0.90377

N -3.35359 -5.22028 1.81982

C 4.20583 -3.21034 -0.14405

H 3.72396 -4.01600 -0.72230

H 4.79687 -3.66022 0.66695

H 4.89568 -2.70460 -0.84072

C 3.17708 -2.22606 0.39910

C 3.18426 -2.04350 1.81041

H 3.86531 -2.70816 2.34824

C 2.61943 -1.01366 2.61012

C 3.10581 -0.95628 4.05326

H 3.84849 -0.15071 4.18636

H 3.58164 -1.90435 4.34653

H 2.27655 -0.73683 4.74539

C 1.46776 1.04810 2.99187

C 2.39510 2.13331 3.01818

C 2.08354 3.27229 3.78446

H 2.78981 4.11003 3.80189

C 0.89260 3.36218 4.51508

H 0.67157 4.25757 5.10617

C -0.00939 2.29288 4.48549

H -0.93964 2.35450 5.06239

C 0.25709 1.12787 3.73951

C -0.74940 -0.02203 3.77314

H -0.31923 -0.85203 3.18769

C -0.98709 -0.54052 5.20976

H -0.04325 -0.84802 5.69190

H -1.66451 -1.41280 5.19842

H -1.45021 0.23145 5.84960

C -2.08529 0.37487 3.10578

H -2.55202 1.22823 3.63073

H -2.79830 -0.46836 3.13141

H -1.93789 0.65763 2.04970

C 3.69631 2.11653 2.21209

H 3.82135 1.10431 1.79151

C 4.93692 2.41925 3.08284

H 5.85910 2.31379 2.48503

H 5.01353 1.73816 3.94799

H 4.91698 3.45129 3.47545

C 3.61813 3.10501 1.02705

H 2.76326 2.88306 0.36842

H 4.54457 3.06246 0.42650

H 3.49398 4.14241 1.38317

C 2.56439 -1.75914 -1.86438

C 3.37892 -0.82332 -2.56871

C 3.58503 -1.00548 -3.94976

H 4.21911 -0.29405 -4.49062

C 2.99565 -2.06742 -4.64441

H 3.17204 -2.19300 -5.71811

C 2.16893 -2.96039 -3.95331

H 1.69228 -3.78483 -4.49629

C 1.93689 -2.82969 -2.56963

C 0.99972 -3.82590 -1.88431

H 1.00779 -3.60013 -0.80496

C -0.45172 -3.65202 -2.38853

H -0.80322 -2.61538 -2.26127

H -1.13823 -4.31806 -1.83662

H -0.52936 -3.90005 -3.46224

C 1.44981 -5.29349 -2.06637

H 1.41652 -5.59853 -3.12723

H 0.78189 -5.97206 -1.50672

H 2.47971 -5.46054 -1.70741

C 4.03089 0.37361 -1.87387

H 3.65194 0.40055 -0.83692

C 5.56914 0.23103 -1.80899

H 5.87522 -0.68147 -1.26962

H 6.01706 1.09644 -1.28932

H 6.00784 0.18465 -2.82182

C 3.63735 1.70284 -2.55520

H 4.01841 1.75688 -3.59050

H 4.05759 2.55916 -2.00088

H 2.54294 1.82481 -2.59061

C -0.23890 -3.22918 1.60432

H 0.81352 -3.26155 1.91294

C -1.09899 -4.27288 1.93030

H -0.70801 -5.12051 2.49626

C -2.46397 -4.21546 1.52183

C -2.84951 -3.05681 0.78936

H -3.87262 -2.92342 0.43315

C -1.91420 -2.06301 0.51077

H -2.19666 -1.15823 -0.04105

C -4.73920 -5.12627 1.36688

H -5.24482 -4.23513 1.78433

H -5.28879 -6.01675 1.70212

H -4.80714 -5.07629 0.26349

C -2.90895 -6.39504 2.56524

H -2.11683 -6.94858 2.02567

H -3.76077 -7.07419 2.70839

H -2.51952 -6.12119 3.56399

C -1.03114 1.68875 -1.24839

C -1.03732 1.45796 -2.78596

C -0.40964 0.32662 -3.33620

H 0.10889 -0.35908 -2.66149

C -0.43331 0.08438 -4.72034

H 0.07884 -0.79843 -5.11815

C -1.09996 0.96782 -5.58256

H -1.12276 0.77939 -6.66165

C -1.73905 2.09966 -5.04643

H -2.26154 2.80050 -5.70787

C -1.70949 2.34193 -3.66470

H -2.20925 3.22728 -3.25694

C -0.34002 3.02931 -0.87732

C -0.44274 3.48564 0.45558

H -0.99875 2.88578 1.18487

C 0.14827 4.68862 0.86343

H 0.04557 5.01511 1.90353

C 0.87395 5.46570 -0.05669

H 1.33195 6.41059 0.25614

C 1.00706 5.01572 -1.37790

H 1.57756 5.60514 -2.10488

C 0.40491 3.81044 -1.78298

H 0.50187 3.48563 -2.82329

C -4.69751 2.65878 -0.51315

C -4.77994 1.08738 -0.67140

C -5.59091 0.37187 0.41284

H -5.57777 -0.71616 0.23188

H -6.64394 0.70229 0.39542

H -5.18015 0.55675 1.41649

C -5.23960 0.64124 -2.07066

H -4.64849 1.13478 -2.85926

H -6.30794 0.85920 -2.23775

H -5.08820 -0.44617 -2.16879

C -4.74853 3.13009 0.95099

H -4.03624 2.56698 1.57585

H -5.75864 3.02101 1.38109

H -4.46611 4.19430 0.99136

C -5.69843 3.45023 -1.36099

H -5.53454 4.53018 -1.21285

H -6.73605 3.21941 -1.06305

H -5.58332 3.23295 -2.43328

B -2.60193 1.78569 -0.90601

**14**

SCF (BP86) Energy = -2418.47374141

Enthalpy 0K = -2417.319202

Enthalpy 298K = -2417.247675

Free Energy 298K = -2417.427488

Lowest Frequency = 15.4633 cm^-1^

Second Frequency = 16.4524 cm^-1^

SCF (Toluene) Energy = -2418.480379

SCF (BP86-D3) Energy = -2418.817935

SCF (BS2) Energy = -2618.30321412

C -1.28709 -1.54818 -4.28094

H -0.31190 -1.99158 -4.55567

H -1.66091 -0.99533 -5.15651

H -1.97071 -2.38188 -4.06178

C -1.09994 -0.63175 -3.07525

C -0.84716 0.73338 -3.38186

H -0.76748 0.94445 -4.45295

C -0.84370 1.89359 -2.56372

C -0.77265 3.21297 -3.33085

H -1.06152 4.07188 -2.70864

H -1.40490 3.19144 -4.23296

H 0.26779 3.37838 -3.66718

C -1.20387 3.08214 -0.49752

C -2.57728 3.47896 -0.38438

C -2.90295 4.59005 0.41901

H -3.95234 4.89604 0.50124

C -1.92188 5.31198 1.10803

H -2.19871 6.16894 1.73173

C -0.58118 4.93606 0.97271

H 0.19569 5.51497 1.48493

C -0.19645 3.84154 0.17340

C 1.29331 3.56388 -0.02622

H 1.38969 2.59586 -0.54857

C 2.08031 3.46405 1.29666

H 2.11456 4.43163 1.82987

H 3.11971 3.15072 1.10039

H 1.63331 2.71556 1.96888

C 1.91650 4.65502 -0.93068

H 1.40669 4.71787 -1.90629

H 2.98428 4.44501 -1.11608

H 1.84485 5.64844 -0.45204

C -3.70566 2.76302 -1.13474

H -3.28554 1.83959 -1.56848

C -4.24344 3.63461 -2.29563

H -4.69588 4.56590 -1.90977

H -5.02155 3.09163 -2.86101

H -3.44664 3.91896 -3.00061

C -4.87627 2.36006 -0.21063

H -5.36534 3.24269 0.23890

H -4.54455 1.70180 0.60858

H -5.64787 1.82185 -0.78890

C -1.65861 -2.45218 -1.60640

C -3.06418 -2.61904 -1.39188

C -3.56416 -3.90819 -1.12546

H -4.64092 -4.04122 -0.97420

C -2.71792 -5.02078 -1.04060

H -3.12842 -6.01449 -0.83008

C -1.34284 -4.84670 -1.22722

H -0.67443 -5.71327 -1.16198

C -0.79109 -3.58164 -1.51484

C 0.71598 -3.46881 -1.73965

H 0.94458 -2.40674 -1.93419

C 1.51764 -3.88329 -0.48710

H 1.23128 -3.27598 0.38610

H 2.59857 -3.73312 -0.65219

H 1.35732 -4.94723 -0.23423

C 1.16852 -4.29676 -2.96457

H 1.00154 -5.37690 -2.80227

H 2.24740 -4.15376 -3.15322

H 0.62030 -4.01072 -3.87784

C -4.02874 -1.43230 -1.45338

H -3.42203 -0.52279 -1.29550

C -5.10623 -1.48174 -0.34881

H -5.81703 -2.31371 -0.50165

H -5.69619 -0.54961 -0.35312

H -4.65605 -1.59838 0.65089

C -4.70449 -1.31707 -2.84041

H -3.96666 -1.18079 -3.64659

H -5.39555 -0.45548 -2.86614

H -5.28984 -2.22668 -3.06685

C -1.87034 -1.59922 1.71728

H -1.36375 -2.40034 1.16896

C -2.65016 -1.91427 2.82809

H -2.73681 -2.96008 3.12812

C -3.31616 -0.87588 3.54080

C -3.12767 0.44329 3.03819

H -3.59422 1.30823 3.51318

C -2.32547 0.64836 1.91734

H -2.17831 1.66416 1.53476

C -4.75159 -0.03218 5.34553

H -4.02005 0.69491 5.74682

H -5.32768 -0.43646 6.18941

H -5.45137 0.51217 4.68353

C -4.24737 -2.50164 5.12831

H -4.70955 -3.15479 4.36402

H -4.89925 -2.50010 6.01290

H -3.27606 -2.94502 5.41946

C 2.42527 -0.17152 0.16763

C 3.01513 0.12911 -2.15528

H 3.97972 -0.31815 -1.81830

C 3.28467 1.61555 -2.46867

H 2.35417 2.10578 -2.80338

H 4.03984 1.72672 -3.26862

H 3.64955 2.14125 -1.57117

C 2.60429 -0.61472 -3.43710

H 2.51206 -1.69778 -3.25815

H 3.35285 -0.46157 -4.23486

H 1.63083 -0.23548 -3.79332

C 1.85038 -0.44781 2.50290

H 2.85795 -0.00092 2.67103

C 1.95239 -1.95096 2.84676

H 0.97001 -2.43799 2.71236

H 2.26582 -2.10268 3.89672

H 2.68096 -2.45477 2.19128

C 0.89297 0.24849 3.48604

H 0.78457 1.31988 3.25163

H 1.27063 0.15925 4.52015

H -0.10874 -0.21064 3.45244

C 6.13626 0.44223 0.95620

C 6.02891 -1.13335 1.10745

C 7.07695 -1.93226 0.32578

H 6.89258 -3.01093 0.45782

H 8.09357 -1.71281 0.69522

H 7.03853 -1.71058 -0.75096

C 5.98300 -1.60957 2.56959

H 5.21804 -1.06467 3.14611

H 6.95808 -1.48158 3.06900

H 5.72205 -2.67996 2.58891

C 6.78022 0.89265 -0.36631

H 6.30441 0.40539 -1.23265

H 7.86065 0.67177 -0.38743

H 6.64735 1.98093 -0.47623

C 6.79741 1.16283 2.13572

H 6.78582 2.25047 1.95732

H 7.84917 0.84768 2.24812

H 6.26886 0.96810 3.08057

N -1.13519 -1.13335 -1.82807

N -0.86279 1.88518 -1.21523

N -1.68615 -0.34039 1.24266

N -4.09341 -1.13196 4.64532

N 1.45159 -0.22370 1.10676

N 2.00587 -0.02907 -1.09943

O 4.72711 0.85847 0.89827

O 4.70840 -1.42670 0.53090

Mg -0.12976 0.05638 -0.34887

B 3.97906 -0.25060 0.54009

**11·IMe**

SCF (BP86) Energy = -2085.10769048

Enthalpy 0K = -2084.117588

Enthalpy 298K = -2084.052648

Free Energy 298K = -2084.225001

Lowest Frequency = 5.0720 cm^-1^

Second Frequency = 15.5714 cm^-1^

SCF (Toluene) Energy = -2085.11529448

SCF (BP86-D3BJ) Energy = -2085.395631

SCF (BS2) Energy = -2569.24253183

Mg -0.83410 -0.12361 0.32945

O 1.43438 -1.82320 -1.55529

O 0.05777 -0.45741 -2.79124

N -0.29734 0.21978 2.35502

N -2.64513 -1.08894 0.86858

N -1.55161 1.88766 -0.26296

N -2.82156 5.73876 -1.47964

C -0.87656 0.66630 4.73000

H -0.13651 -0.03548 5.15193

H -1.75801 0.67830 5.38759

H -0.40227 1.66129 4.75273

C -1.24810 0.25593 3.30983

C -2.60940 -0.10086 3.10781

H -3.24838 0.05067 3.98165

C -3.23150 -0.81745 2.04742

C -4.63474 -1.33787 2.33524

H -5.29838 -1.20545 1.46557

H -5.07501 -0.83208 3.20783

H -4.61023 -2.42119 2.54772

C 1.07099 0.45752 2.74099

C 1.92018 -0.65260 3.03004

C 1.42975 -2.09994 2.95562

H 0.38867 -2.07863 2.58825

C 2.26766 -2.92971 1.95607

H 2.19537 -2.52544 0.93281

H 1.91073 -3.97389 1.93237

H 3.33359 -2.95320 2.24444

C 1.42118 -2.76847 4.35013

H 1.02585 -3.79737 4.28368

H 0.79509 -2.21104 5.06768

H 2.44009 -2.82899 4.77245

C 3.25670 -0.40890 3.40467

H 3.90522 -1.25991 3.64171

C 3.77220 0.89044 3.48221

H 4.81213 1.05788 3.78201

C 2.94331 1.97227 3.16165

H 3.34735 2.99037 3.20268

C 1.59796 1.78420 2.78953

C 0.76024 3.00876 2.41834

H -0.27678 2.66576 2.26592

C 0.74493 4.08762 3.52466

H 1.74894 4.51692 3.68879

H 0.39616 3.68465 4.49070

H 0.07440 4.91829 3.24168

C 1.25019 3.62018 1.08552

H 0.60372 4.46133 0.77845

H 1.24128 2.87118 0.27670

H 2.28222 4.00223 1.18145

C -3.27787 -2.03167 -0.01885

C -3.05796 -3.42964 0.16814

C -2.17047 -3.97259 1.28970

H -1.88309 -3.12344 1.93307

C -0.87272 -4.57670 0.70593

H -0.22294 -4.95742 1.51371

H -0.30454 -3.82725 0.12995

H -1.09893 -5.42214 0.03177

C -2.90073 -5.00727 2.17515

H -3.16825 -5.91467 1.60521

H -3.83235 -4.59815 2.60240

H -2.25335 -5.32272 3.01208

C -3.64181 -4.33846 -0.73503

H -3.47072 -5.41196 -0.59404

C -4.42960 -3.90233 -1.80644

H -4.87881 -4.62607 -2.49504

C -4.63001 -2.53034 -1.99049

H -5.24023 -2.18216 -2.83200

C -4.06474 -1.57928 -1.11828

C -4.31137 -0.09508 -1.38705

H -3.77802 0.47295 -0.60650

C -5.81007 0.27197 -1.29311

H -5.95463 1.35533 -1.45354

H -6.23310 0.01591 -0.30660

H -6.40412 -0.25994 -2.05743

C -3.73215 0.32589 -2.75703

H -2.66008 0.07903 -2.83478

H -3.85028 1.41341 -2.91016

H -4.25569 -0.18620 -3.58443

C -2.34691 2.66039 0.52306

H -2.63876 2.21872 1.48412

C -2.79393 3.92971 0.16943

H -3.43364 4.47642 0.86472

C -2.41363 4.48699 -1.08725

C -1.59039 3.66768 -1.91259

H -1.26048 4.00230 -2.89778

C -1.19766 2.40817 -1.46742

H -0.58227 1.75426 -2.09795

C -3.68704 6.53295 -0.61078

H -4.65587 6.03113 -0.42520

H -3.89108 7.49917 -1.09239

H -3.20865 6.73344 0.36628

C -2.42416 6.26090 -2.78538

H -1.32385 6.32032 -2.88058

H -2.83020 7.27466 -2.90614

H -2.80988 5.63551 -3.61297

B 0.39956 -0.87443 -1.48907

C 1.60680 -2.25157 -2.95592

C 1.00283 -1.04025 -3.76046

C 3.09311 -2.52511 -3.20673

H 3.43099 -3.35346 -2.56240

H 3.26450 -2.82244 -4.25660

H 3.72282 -1.64942 -2.98750

C 0.80037 -3.55393 -3.11424

H 1.16388 -4.28908 -2.37808

H -0.27182 -3.38622 -2.92351

H 0.91807 -3.98827 -4.12205

C 0.22157 -1.42255 -5.02255

H 0.87963 -1.91490 -5.76032

H -0.61556 -2.09807 -4.79272

H -0.19308 -0.51500 -5.49250

C 2.03234 0.05468 -4.09242

H 1.50134 0.94384 -4.47156

H 2.60396 0.35285 -3.19912

H 2.74578 -0.27443 -4.86722

C 3.91444 0.47743 -0.32958

H 4.00137 0.43152 0.76176

H 3.54250 1.45293 -0.66193

H 3.31221 -0.34858 -0.72821

I 5.93936 0.27712 -1.14227

**TS(11-12)a**

SCF (BP86) Energy = -2085.08794452

Enthalpy 0K = -2084.098424

Enthalpy 298K = -2084.034201

Free Energy 298K = -2084.201441

Lowest Frequency = -415.9157 cm^-1^

Second Frequency = 10.0474 cm^-1^

SCF (Toluene) Energy = -2085.09995526

SCF (BP86-D3BJ) Energy = -2085.387917

SCF (BS2) Energy = -2569.21943766

Mg 0.41676 0.08843 -0.48194

O 1.20954 -1.95776 2.14678

O 0.73693 0.20969 2.77759

N -0.46252 -0.64631 -2.24421

N 2.26136 0.51541 -1.41153

N -0.50717 2.04962 -0.22506

N -2.46756 5.78203 0.15025

C -0.68777 -0.69971 -4.71716

H -0.75019 -1.80012 -4.76775

H -0.16115 -0.33757 -5.61197

H -1.72633 -0.33188 -4.74362

C 0.02468 -0.26893 -3.44227

C 1.20782 0.49906 -3.62468

H 1.40335 0.78386 -4.66160

C 2.28249 0.76737 -2.73566

C 3.54555 1.32348 -3.38155

H 4.01455 2.09617 -2.75214

H 3.32643 1.74754 -4.37309

H 4.29817 0.52682 -3.51449

C -1.59740 -1.54248 -2.21121

C -1.37179 -2.94140 -2.04149

C 0.03302 -3.54504 -1.96570

H 0.75764 -2.71116 -1.96760

C 0.24417 -4.35224 -0.66494

H 0.14292 -3.71635 0.22956

H 1.25521 -4.79436 -0.64798

H -0.48117 -5.17997 -0.57914

C 0.33195 -4.42342 -3.20355

H 1.36111 -4.82149 -3.15859

H 0.22924 -3.85465 -4.14346

H -0.35829 -5.28386 -3.25749

C -2.48335 -3.80255 -1.96039

H -2.31469 -4.87780 -1.83405

C -3.79255 -3.31663 -2.02960

H -4.64264 -4.00141 -1.94928

C -4.00468 -1.94349 -2.18494

H -5.02983 -1.55943 -2.22127

C -2.93134 -1.03693 -2.28308

C -3.24648 0.45272 -2.44377

H -2.28997 0.98159 -2.59788

C -4.14649 0.73653 -3.66936

H -5.14809 0.28969 -3.54447

H -3.72119 0.33387 -4.60455

H -4.28576 1.82435 -3.80021

C -3.90461 1.02810 -1.16905

H -4.08857 2.11136 -1.28325

H -3.27379 0.87803 -0.27884

H -4.87075 0.53683 -0.96333

C 3.50599 0.56902 -0.68509

C 4.38269 -0.55835 -0.70777

C 4.06718 -1.82719 -1.50408

H 3.14585 -1.64263 -2.08247

C 3.79350 -3.01864 -0.55800

H 3.53450 -3.92183 -1.13849

H 2.96515 -2.80274 0.13643

H 4.68843 -3.25691 0.04489

C 5.18854 -2.18651 -2.50581

H 6.12843 -2.44461 -1.98653

H 5.41032 -1.35456 -3.19583

H 4.89564 -3.06120 -3.11233

C 5.56928 -0.51169 0.04909

H 6.24213 -1.37675 0.03594

C 5.90593 0.60967 0.81633

H 6.83547 0.62606 1.39533

C 5.04162 1.70989 0.83307

H 5.30436 2.59349 1.42619

C 3.84173 1.71562 0.09386

C 2.95682 2.96209 0.13499

H 2.06018 2.74835 -0.47096

C 3.66308 4.18894 -0.48778

H 2.99259 5.06666 -0.47998

H 3.96504 4.00301 -1.53247

H 4.57250 4.45816 0.07816

C 2.49074 3.28113 1.57302

H 1.97568 2.41935 2.02851

H 1.79678 4.14028 1.57296

H 3.34549 3.54604 2.22117

C -0.73883 2.84916 -1.30158

H -0.38851 2.46369 -2.26683

C -1.36848 4.08657 -1.23060

H -1.51129 4.65746 -2.14975

C -1.82091 4.57872 0.02986

C -1.55543 3.74810 1.15742

H -1.84894 4.04487 2.16564

C -0.90998 2.52891 0.98300

H -0.67398 1.89721 1.84703

C -2.72545 6.59733 -1.03531

H -1.78579 6.88898 -1.54072

H -3.24688 7.51582 -0.73330

H -3.36314 6.06564 -1.76658

C -2.93777 6.23208 1.46010

H -3.65618 5.51690 1.90217

H -3.44552 7.19955 1.34638

H -2.09959 6.36684 2.16917

B 0.62914 -0.75057 1.76261

C 1.89542 -1.76872 3.44009

C 1.22089 -0.45580 4.00686

C 1.66313 -3.02824 4.28204

H 2.12615 -3.89520 3.78311

H 2.12507 -2.92422 5.27940

H 0.59212 -3.24299 4.41297

C 3.39232 -1.60634 3.13097

H 3.74578 -2.49891 2.59077

H 3.58448 -0.72978 2.49220

H 3.98617 -1.50817 4.05576

C 2.17590 0.50153 4.72640

H 2.59862 0.02646 5.62884

H 3.00426 0.81730 4.07529

H 1.62671 1.40258 5.04635

C -0.01956 -0.71674 4.87870

H -0.52433 0.24161 5.08348

H -0.74702 -1.37756 4.38071

H 0.25589 -1.17046 5.84580

C -1.61387 -1.09995 1.45884

H -1.88318 -1.16966 0.40452

H -1.68623 -0.15689 1.98718

H -1.24528 -1.99233 1.95552

I -4.03656 -1.66298 2.30035

**INT(11-12)**

SCF (BP86) Energy = -2085.18960256

Enthalpy 0K = -2084.196789

Enthalpy 298K = -2084.133088

Free Energy 298K = -2084.298239

Lowest Frequency = 10.1926 cm^-1^

Second Frequency = 18.5663 cm^-1^

SCF (Toluene) Energy = -2085.20898786

SCF (BP86-D3BJ) Energy = -2085.497967

SCF (BS2) Energy = -2569.31946662

Mg -0.64609 0.19951 -0.24516

O 0.77017 -0.41563 3.81312

O -0.12194 -0.51066 1.67496

N -1.69832 2.01186 -0.38418

N -1.86249 -0.85346 -1.61622

N 1.20602 0.55250 -1.20574

N 4.83835 1.22910 -3.27028

C -3.00824 3.61710 -1.78932

H -3.10907 4.21331 -0.87166

H -4.00145 3.49184 -2.24884

H -2.39200 4.19465 -2.50058

C -2.37199 2.25656 -1.52406

C -2.55926 1.30796 -2.56507

H -3.04886 1.71944 -3.45333

C -2.43234 -0.10360 -2.58789

C -3.04879 -0.75914 -3.82177

H -2.73878 -1.80581 -3.94386

H -2.76390 -0.19188 -4.72337

H -4.15038 -0.73507 -3.76429

C -1.59963 3.03842 0.62529

C -2.38386 2.89919 1.81064

C -3.43186 1.79086 1.93397

H -3.07512 0.93007 1.33635

C -3.65246 1.30467 3.37982

H -2.69987 1.06448 3.88109

H -4.28664 0.40133 3.38413

H -4.17187 2.06095 3.99436

C -4.77796 2.23790 1.31422

H -5.52805 1.43073 1.38723

H -4.66815 2.50475 0.25103

H -5.17360 3.12086 1.84660

C -2.22348 3.83965 2.84519

H -2.81977 3.74150 3.75758

C -1.31735 4.90116 2.72934

H -1.20215 5.61983 3.54731

C -0.56648 5.03857 1.55849

H 0.13752 5.87312 1.46682

C -0.68614 4.12735 0.48915

C 0.16540 4.36441 -0.76367

H -0.09616 3.58977 -1.50501

C -0.12521 5.74879 -1.39288

H 0.20996 6.56552 -0.72995

H -1.19916 5.90208 -1.58713

H 0.41749 5.85853 -2.34823

C 1.67974 4.23721 -0.47772

H 2.25833 4.42668 -1.39853

H 1.94957 3.23457 -0.11189

H 2.00583 4.97105 0.27982

C -2.10126 -2.27746 -1.63465

C -3.38769 -2.79023 -1.27513

C -4.60158 -1.89799 -0.98030

H -4.31777 -0.85022 -1.17511

C -5.05182 -1.98388 0.49618

H -5.94772 -1.35965 0.66117

H -4.26471 -1.63634 1.18313

H -5.31045 -3.02004 0.77695

C -5.79666 -2.24521 -1.90145

H -6.20676 -3.24336 -1.66785

H -5.51293 -2.24881 -2.96656

H -6.61263 -1.51443 -1.76398

C -3.55918 -4.18757 -1.19834

H -4.53839 -4.58699 -0.91095

C -2.51430 -5.07111 -1.48272

H -2.66979 -6.15285 -1.41374

C -1.26886 -4.56101 -1.86895

H -0.45770 -5.25465 -2.10823

C -1.03455 -3.17517 -1.95758

C 0.32260 -2.66211 -2.45213

H 0.54822 -1.72931 -1.90374

C 0.26816 -2.28680 -3.95345

H 1.25517 -1.92667 -4.29179

H -0.46686 -1.49247 -4.15432

H -0.00166 -3.16677 -4.56408

C 1.49527 -3.63465 -2.21206

H 1.54460 -3.99013 -1.17038

H 2.44868 -3.12751 -2.43305

H 1.43555 -4.51816 -2.87255

C 1.23075 1.13520 -2.44083

H 0.25593 1.40861 -2.86317

C 2.39428 1.37928 -3.15915

H 2.31449 1.84976 -4.14117

C 3.66118 1.01081 -2.60703

C 3.62941 0.39669 -1.32340

H 4.51884 0.05195 -0.78555

C 2.41646 0.19503 -0.68424

H 2.44549 -0.29239 0.30068

C 4.83542 1.85797 -4.58823

H 4.26937 1.25888 -5.32689

H 5.87014 1.94801 -4.94592

H 4.39574 2.87302 -4.55587

C 6.10564 0.82438 -2.65215

H 6.27017 1.34406 -1.69133

H 6.93107 1.07912 -3.33093

H 6.13326 -0.26322 -2.46074

B 0.64054 0.23741 2.63040

C 0.13914 -1.73013 3.77078

C -0.27168 -1.91982 2.24094

C 1.17761 -2.74710 4.26358

H 1.41396 -2.52963 5.31765

H 0.78238 -3.77591 4.20866

H 2.11507 -2.67678 3.68719

C -1.04794 -1.67031 4.74713

H -0.66345 -1.39881 5.74298

H -1.79092 -0.91129 4.45466

H -1.55483 -2.64580 4.83037

C -1.71972 -2.34911 2.03151

H -1.88277 -3.34301 2.48071

H -2.42968 -1.64535 2.49210

H -1.94857 -2.44385 0.95775

C 0.68731 -2.79850 1.44026

H 0.37492 -2.81001 0.38270

H 1.73615 -2.45263 1.51183

H 0.64119 -3.83950 1.80012

C 1.27392 1.63900 2.35876

H 1.15038 2.29446 3.23601

H 0.88011 2.17224 1.48186

H 2.36082 1.48388 2.22001

I 4.47094 -1.42706 1.93584

**TS(11-12)b**

SCF (BP86) Energy = -2085.17765432

Enthalpy 0K = -2084.184769

Enthalpy 298K = -2084.122203

Free Energy 298K = -2084.282246

Lowest Frequency = -30.6424 cm^-1^

Second Frequency = 15.5610 cm^-1^

SCF (Toluene) Energy = -2085.191095

SCF (BP86-D3) Energy = -2085.493627

SCF (BS2) Energy = -2569.310411

Mg -0.03106 0.03956 -0.06623

O -1.81141 1.06336 3.75948

O -1.31063 0.20389 1.66478

N -0.18323 1.93433 -1.15193

N -0.83953 -1.13253 -1.65019

N 2.13474 -0.21132 -0.37228

N 6.29179 -0.82313 -0.98303

C 0.17740 3.02703 -3.38905

H 0.03510 3.98137 -2.86635

H -0.52630 2.97767 -4.23636

H 1.19338 3.01124 -3.82075

C -0.00802 1.81211 -2.47924

C 0.01660 0.58083 -3.19596

H 0.31674 0.69917 -4.24158

C -0.43226 -0.71949 -2.87951

C -0.36103 -1.71060 -4.04455

H -1.12571 -2.49620 -3.98475

H 0.62223 -2.21743 -4.03011

H -0.44733 -1.19791 -5.01457

C -0.28927 3.27320 -0.60723

C -1.58759 3.81298 -0.35235

C -2.85931 3.04574 -0.72232

H -2.63292 1.96918 -0.61332

C -4.06112 3.38011 0.18555

H -3.80441 3.31410 1.25666

H -4.89331 2.68438 -0.01690

H -4.44658 4.39777 -0.00319

C -3.25493 3.28509 -2.19966

H -4.18312 2.73852 -2.44462

H -2.47232 2.94620 -2.89475

H -3.43483 4.35917 -2.38410

C -1.69261 5.10169 0.20592

H -2.68394 5.52051 0.40310

C -0.55819 5.86332 0.50798

H -0.66233 6.86436 0.93960

C 0.70744 5.33195 0.24875

H 1.59763 5.92785 0.47866

C 0.87441 4.04521 -0.30278

C 2.30617 3.56141 -0.56034

H 2.25560 2.50787 -0.88515

C 2.99789 4.38035 -1.67823

H 3.11170 5.43647 -1.37534

H 2.43240 4.36612 -2.62213

H 4.00767 3.98044 -1.87794

C 3.18393 3.63132 0.71247

H 4.16920 3.17399 0.51840

H 2.72672 3.10874 1.56700

H 3.35897 4.67721 1.02034

C -1.62692 -2.34566 -1.61811

C -2.99157 -2.29806 -2.05789

C -3.67511 -0.99845 -2.50930

H -3.08979 -0.15854 -2.09127

C -5.12968 -0.88191 -1.99366

H -5.50725 0.14220 -2.15942

H -5.21528 -1.11173 -0.92019

H -5.80799 -1.56385 -2.53615

C -3.69350 -0.83004 -4.04887

H -4.18821 -1.69438 -4.52696

H -2.68422 -0.73567 -4.47370

H -4.25877 0.07738 -4.32607

C -3.73822 -3.49250 -2.07703

H -4.77698 -3.46583 -2.42280

C -3.18637 -4.70861 -1.66030

H -3.78435 -5.62580 -1.68721

C -1.86917 -4.73686 -1.19304

H -1.44115 -5.68382 -0.84873

C -1.06914 -3.57665 -1.15687

C 0.37107 -3.70021 -0.65299

H 0.73214 -2.68990 -0.38658

C 1.30693 -4.25731 -1.75216

H 2.34573 -4.30697 -1.38236

H 1.29632 -3.63726 -2.66341

H 0.99881 -5.27767 -2.04276

C 0.49526 -4.57248 0.61447

H -0.20983 -4.25695 1.39835

H 1.50790 -4.47337 1.03722

H 0.31992 -5.64118 0.39429

C 2.62053 -0.84366 -1.47408

H 1.87424 -1.19040 -2.19101

C 3.96971 -1.07568 -1.72051

H 4.24975 -1.61600 -2.62630

C 4.94652 -0.62403 -0.78710

C 4.44121 0.04405 0.36238

H 5.10084 0.40127 1.15436

C 3.07009 0.20565 0.52741

H 2.69089 0.66656 1.44150

C 6.75484 -1.59914 -2.13072

H 6.40646 -2.65001 -2.09490

H 7.85374 -1.60145 -2.14303

H 6.40805 -1.15141 -3.07973

C 7.24187 -0.44995 0.06410

H 7.18283 0.63041 0.28893

H 8.26177 -0.66620 -0.28333

H 7.06731 -1.00986 1.00287

B -0.95996 1.11466 2.69378

C -2.66094 -0.12230 3.66065

C -2.53257 -0.56053 2.13284

C -2.10369 -1.14635 4.66201

H -2.11361 -0.69067 5.66566

H -2.72339 -2.05791 4.69269

H -1.06336 -1.42225 4.41941

C -4.07996 0.29219 4.07648

H -4.06063 0.61980 5.12822

H -4.46846 1.12365 3.46921

H -4.77754 -0.55893 3.99985

C -3.68175 -0.07661 1.25075

H -4.61183 -0.61603 1.49531

H -3.86356 1.00169 1.36283

H -3.43846 -0.28339 0.19757

C -2.28847 -2.05341 1.93803

H -2.24656 -2.30294 0.86614

H -1.34807 -2.36920 2.41617

H -3.12764 -2.62290 2.37329

C 0.21444 2.14783 2.60130

H -0.16041 3.18200 2.69461

H 0.79068 2.10035 1.66462

H 0.91166 1.95769 3.43446

I 1.49409 -1.56053 2.96994
